# Macrophage Extracellular Traps in Immunity and Cancer

**DOI:** 10.1002/advs.202520421

**Published:** 2026-02-16

**Authors:** Junyao Li, Eugenie Nepovimova, Adam Grundel, Lukas Peter, Qinghua Wu, Kamil Kuca

**Affiliations:** ^1^ College of Life Science Yangtze University Jingzhou China; ^2^ Department of Chemistry Faculty of Science University of Hradec Králové Hradec Králové Czech Republic; ^3^ Faculty of Materials Science and Technologies VSB‐Technical University of Ostrava Ostrava‐Poruba Czech Republic; ^4^ Faculty of Informatics and Management University of Hradec Kralove Hradec Kralove Czech Republic; ^5^ Biomedical Research Center University Hospital Hradec Kralove Hradec Kralove Czech Republic

**Keywords:** cancer immunotherapy, immunity, inflammation, macrophage extracellular traps, METs

## Abstract

Macrophage extracellular Traps (METs) are web‐like structures released by activated macrophages, composed of a DNA backbone decorated with various proteins. Their core function lies in entrapping and restricting pathogen dissemination, representing an important innate immune defense mechanism of macrophages. Through the activation of multiple inflammatory factors and protein components, METs amplify the inflammatory cascade and are detected in various inflammatory conditions. Moreover, recognizing chronic inflammation as a major oncogenic driver, the role of METs in cancer is increasingly appreciated. By fostering an immunosuppressive tumor microenvironment and degrading the extracellular matrix, METs facilitate epithelial‐mesenchymal transition, thereby promoting angiogenesis and tumor cell survival. In addition, METs affect the efficacy of cancer immunotherapy. While METs antagonize cancer immunotherapy by creating a physical barrier and fostering immunosuppression, their intrinsic cytotoxicity and ability to amplify inflammatory signals also synergize with immune‐activating strategies. This review comprehensively examines the underlying function of METs in immunity, tumorigenesis, and cancer immunotherapy. Deciphering these functional connections will provide critical insights for targeting METs to enhance the efficacy of cancer immunotherapies.

## Graphical abstract

As a macrophage‐mediated innate defense mechanism, the dysregulated release of METs drives chronic inflammation and influences tumor progression. Furthermore, METs exhibit a functional duality within the tumor microenvironment, capable of both promoting and suppressing tumor development. Simultaneously, they profoundly shape the efficacy of cancer immunotherapy through mechanisms including immunosuppression and tumor antigen presentation.

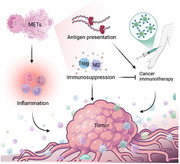

## 1 Introduction

The immune defense system protects against pathogens through a multidimensional mechanism. Macrophages, neutrophils, and adaptive immune cells (T/B lymphocytes) constitute this multilayered defense network [1]. Among the arsenal of innate immune defenses are extracellular traps (ETs), structures composed of a double‐stranded DNA scaffold decorated with various antimicrobial proteins, which are released by immune cells in a process termed “ETosis” [2]. ETs were initially discovered in neutrophils and are now known to be produced by a range of mammalian innate immune cells, including macrophages, eosinophils, and mast cells [2, 3]. Neutrophil extracellular traps (NETs) play a crucial role in clearing infections; however, their excessive formation is closely associated with tissue damage and cancers [4, 5, 6]. Similar to NETs, macrophage extracellular traps (METs) are released by macrophages in response to specific stimuli, and depend on key pathways such as reactive oxygen species (ROS) and peptidyl arginine deiminase (PAD) [7, 8]. While METs play a fundamental role in restricting pathogens and exerting direct killing, their dysregulated presence orchestrates the amplification of inflammatory immune responses and actively promotes cancer progression.

METs potently amplify the inflammatory cascade. This effect stems from the robust inflammatory milieu engendered by METs, which involves the release of cytokines, chemokines, and a spectrum of cytotoxic proteins that collectively disrupt immune homeostasis and drive a feedforward inflammatory cascade. Importantly, chronic inflammation is a well‐established precursor to tumorigenesis [9]. Multiple malignancies, including colorectal and hepatocellular carcinomas, frequently arise from sites of persistent inflammation [10, 11]. These observations suggest that METs may serve as a functional bridge linking inflammatory pathology to cancer initiation and development. Actually, the role of METs within the tumor microenvironment (TME) is gaining growing recognition. METs are identified in several solid tumors, such as colon cancer (CC) and Pancreatic ductal adenocarcinoma (PDAC), where they exhibit potent tumor‐promoting attributes [12, 13]. In most malignancies, elevated MET levels correlate with adverse clinical outcomes, highlighting their potential utility as prognostic biomarkers [12, 14]. Nevertheless, emerging findings point to potential tumor‐suppressive roles for METs in certain malignancies, revealing a layer of functional complexity [15]. A central challenge now lies in delineating the specific mechanisms behind the pro‐versus anti‐tumorigenic effects of METs and in pinpointing the molecular switches that dictate this functional shift.

Cancer immunotherapy encompasses modalities aimed at harnessing the patient's immune system to identify and destroy cancer cells, leveraging approaches such as immune checkpoint blockade, adoptive cell therapy, cytokine treatments, and tumor vaccines [16]. These modalities offer superior specificity and safety profiles compared with conventional treatments and have markedly improved survival in selected populations [17]. The pleiotropic roles of METs in the TME significantly influence immunotherapeutic efficacy. By forming a physical barrier and fostering an immunosuppressive niche, METs hinder drug delivery and the infiltration of immune cells into tumors, thereby compromising the efficacy of cancer immunotherapy. Conversely, when functioning as scaffolds for antigen presentation or instigating a pro‐inflammatory tumor microenvironment, METs may conceivably enhance the response to immunotherapy. Although cancer immunotherapies have achieved remarkable success in hematologic malignancies, their effectiveness in solid tumors remains limited by inadequate immune infiltration and suppressive signaling [18]. Modulating MET activity may offer a promising avenue for enhancing immunotherapy outcomes.

This review synthesizes current understanding of the roles played by METs in immunity, inflammation, and cancer immunotherapy. We examine the triggers and molecular mechanisms governing METs release under inflammatory conditions and detail how they recalibrate immune balance. We further explore the pro‐tumorigenic mechanisms mediated by METs in cancer progression and critically evaluate evidence supporting their context‐dependent anti‐tumor functions. The influence of METs on cancer immunotherapy and potential molecular determinants directing their functional switching are also discussed. This review enhances the understanding of METs in cancer immunotherapy.

## 2 The Structure of METs and Their Release

**FIGURE 1:**
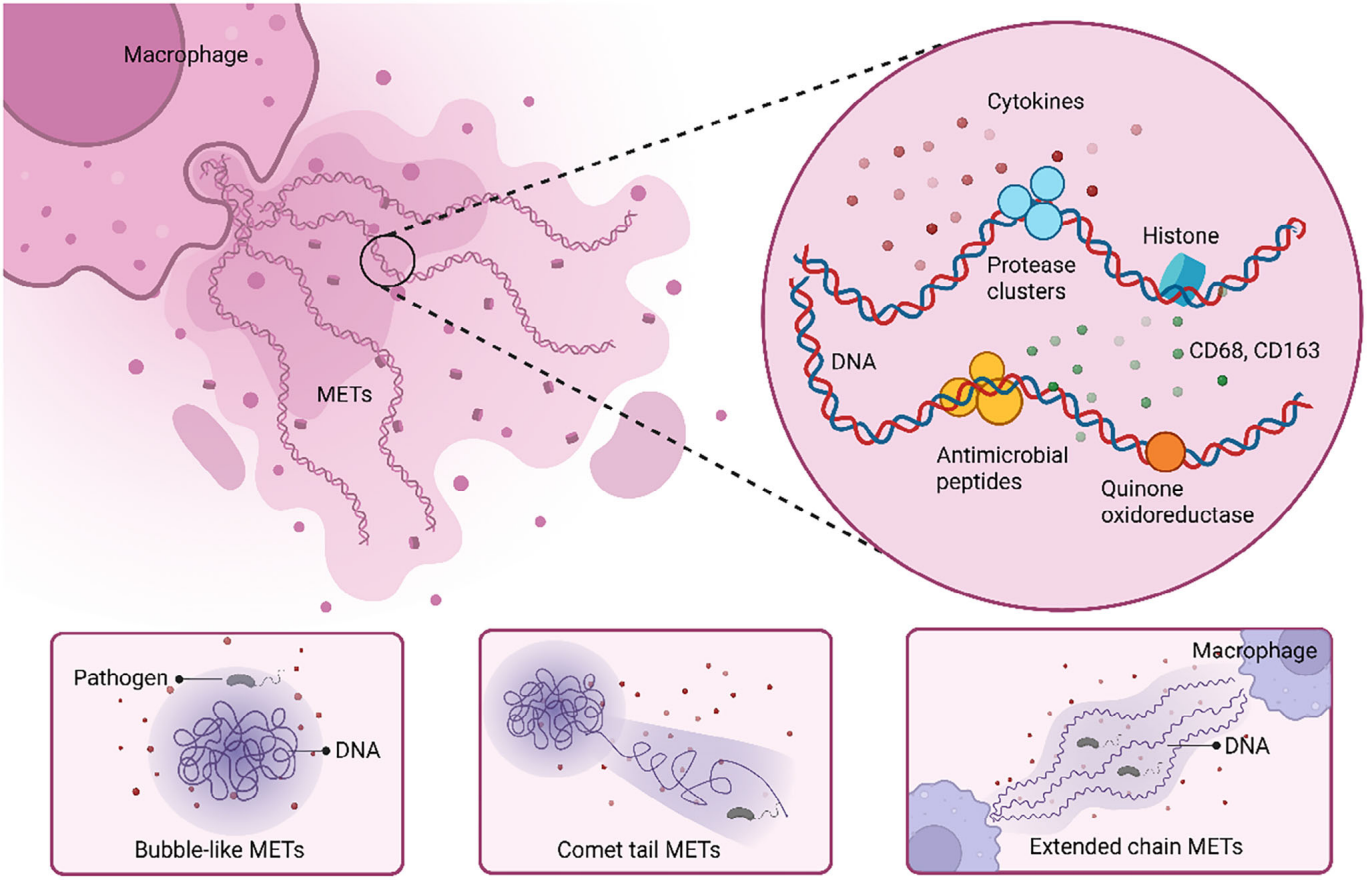
Structural characteristics of METs. The core scaffolding of METs consists of depolymerized chromatin DNA to which a variety of functional proteins are tightly bound, such as histones, antimicrobial peptides, and MMPs. These proteins endow METs with a potent capacity to capture and kill pathogens. The process of METs release is frequently accompanied by the release of various cytokines and macrophage markers such as CD68 and CD163. It is noteworthy that METs morphology is not static but evolves dynamically post‐release, manifesting in distinct forms including diffuse (bubble‐like) structures, spread (comet‐tail) structures, or extended, intercellularly interconnected chain‐like meshwork. Created in https://BioRender.com.

METs are an extracellular defense mechanism of macrophage innate immunity [19]. Their structure consists of a DNA framework, which may originate from mitochondria or the nucleus, adorned with various proteins (Figure 1). Histones are the main protein components in METs [20]. In addition to histones, neutrophil elastase (NE), and myeloperoxidase (MPO), lactoferrin, these include matrix metalloproteinase (MMPs, such as MMP12, MMP9) and quinone oxidoreductase [20, 21, 22]. Notably, quinone oxidoreductase is highly abundant in METs but has seldom been reported in NETs [20]. The DNA scaffold of typical METs forms a complex mesh‐like network that encapsulates pathogens. However, extruded chromatin exhibits heterogeneous and dynamic morphology over time. For example, in PMA‐stimulated crab hyaline cells, the extracellular chromatin exhibited diffuse bubble‐like structures, comet‐tail formations, or extended chains interconnecting adjacent cells [23]. Similarly, METs released by primary mouse peritoneal macrophages in response to *Candida albicans* (*C. albicans*) show the same structural features, with diffuse (bubble‐like) structure evolving into diffuse (comet‐like) structure over time [19]. METs induced by *Mycobacterium tuberculosis* often exhibit linear or net‐like structures [24]. Thus, the distinct structural characteristics of METs contribute significantly to their role as an immune defense against infection.

Various chemical and microbial stimuli induce the formation of METs, though their underlying mechanisms differ significantly (Table 1). Generally, the release of METs increases gradually with prolonged stimulation time and higher doses, eventually reaching a plateau [19]. Pro‐inflammatory cytokines such as IFN‐γ, TNF‐α, and IL‐8 promote METs production by modulating macrophage activation. Additionally, various chemical agents, including lipopolysaccharide (LPS), hypochlorous acid (HOCl), extracellular calcium, palmitate, NE, PS‐MPs, and perfluorooctane sulphonate (PFOS) have been shown to effectively trigger METs release [25, 26, 27, 28, 29, 30, 31, 32, 33, 34, 35, 36, 37, 38, 39, 40]. In terms of pathogens, numerous parasites such as *Schistosoma japonicum* (*S. japonicum*), *Mycoplasma*, and *Neospora caninum*, as well as pathogenic microorganisms including *C. albicans* and *Porphyromonas gingivalis* (*P. gingivalis*), also induce METs release [27, 41, 42, 43, 44, 45, 46, 47, 48, 49, 50, 51, 52, 53, 54]. These pathogens, acting as damage‐associated molecular patterns (DAMPs), are recognized by macrophages and induce METosis. Moreover, METs are detected in diverse pathologies, including atherosclerosis, acute kidney injury, and hepatic ischemia‐reperfusion injury, though their precise roles under these conditions require further study [3, 55, 56]. It is noteworthy that the origin of the chromatin scaffold in METs exhibits a stimulus‐dependent nature. The DNA component of METs can be derived from both nuclear and mitochondrial sources in many contexts, as evidenced by the co‐detection of nuclear and mitochondrial DNA in METs induced by *Mycobacterium massiliense* (*M. mass*) and *C. albicans* [46, 57]. However, the relative abundance of these two DNA sources varies markedly under distinct stimulatory conditions. For instance, TNF‐α induction leads to a more significant increase in nuclear DNA. In contrast, macrophages exposed to nigericin release METs composed predominantly of mitochondrial DNA [20].

**TABLE 1:** A summary of inducers of METs in different cells.

Chemical inducers	Cell model	References	Pathogen inducers	Cell model	References
Biochanin A	Raw264.7 cells	[28]	*Salmonella typhimurium* (*S. Typhimurium*)	Raw264.7 cells	[51]
Perfluorooctane sulphonate (PFOS)	Murine liver macrophages	[36]	*Bacillus licheniformis* (*B. licheniformis*) *Bacillus subtilis* (*B. subtilis*)	Murine macrophage‐like cell line J774A.1	[52]
Hydrogen sulfide (H_2_S)	Chicken tracheal macrophages	[73]	*Clostridium perfringens* (*C. perfringens*)	Peritoneal macrophages of Balb/c mice	[30]
Sulfated vizantin	RAW264.7 cells	[29]	*Salmonella enterica serovar Typhimurium LVR01*	Bone marrow‐derived macrophages (BMDMs) of J774A.1 mice	[47]
Calcium oxalate (CaOx) crystals	RAW264.7 cells, Kidney tissue macrophages	[31]	*Mycoplasma bovis*	Bovine macrophage cell lines (BoMac)	[42]
Platelets	Mouse scleral macrophages, tissue macrophages	[32, 55]	*Uropathogenic Escherichia coli* (*UPEC*)	Suburothelial perivascular macrophages (suPVMs)	[53]
Lonomycin, monosodium urate crystals	Murine macrophages	[39]	*Candida albicans* (*C. albicans*)	Murine macrophage‐like cell line J774A.1	[19]
Polystyrene microplastics (PS‐MPs)	Chicken liver macrophages, RAW264.7 cells	[37, 71]	*Mannheimia haemolytica*	Bovine alveolar macrophages	[49]
HOCl	Human monocyte‐derived macrophages (HMDM)	[26]	*Trichinella spiralis* (*T. spiralis*)	Murine macrophage‐like cell line J774A.1	[173]
PMA	HMDM, BMDMs	[21, 26]	*Streptococcus agalactiae*	Placental macrophages (PMs)	[50]
LPS	HMDM, BMDMs	[21, 38]	*Mycobacterium massiliense* (*M. mass*)	Macrophages differentiated from THP cells	[57]
Extracellular calcium	HMDM	[38]	*Schistosoma japonicum* (*S. japonicum*)	BMDMs	[44]
Neutrophil elastase (NE)	Human blood monocyte derived macrophages (hBMDM), murine BAL Macrophages	[33]	*Giardia lamblia*	Mouse macrophages	[43]
Statins	RAW 264.7 cells	[34]	*Neospora caninum* (*N. caninum*)	Bovine macrophages	[45]
TNF‐α	HMDM	[26]	Aflatoxin B1 (AFB1)	Macrophages differentiated from THP cells, Raw264.7 cells	[61]
IFN‐γ	Primary human macrophages	[27]	*Mayaro virus* (*MAYV*)	Raw264.7 cells	[64]
Cigarette smoke	Alveolar macrophages	[79]	*Sperm*	Macrophages differentiated from THP cells	[70]
NETs	Macrophages differentiated from THP cells	[66]	*Staphylococcus aureus* (*S. aureus*)	BMDMs	[54]
Ambient particulate matter (PM)	BMDMs	[72]	*Porphyromonas gingivalis* (*P. gingivalis*)	Peritoneal macrophages of Balb/c mice	[48]

**FIGURE 2:**
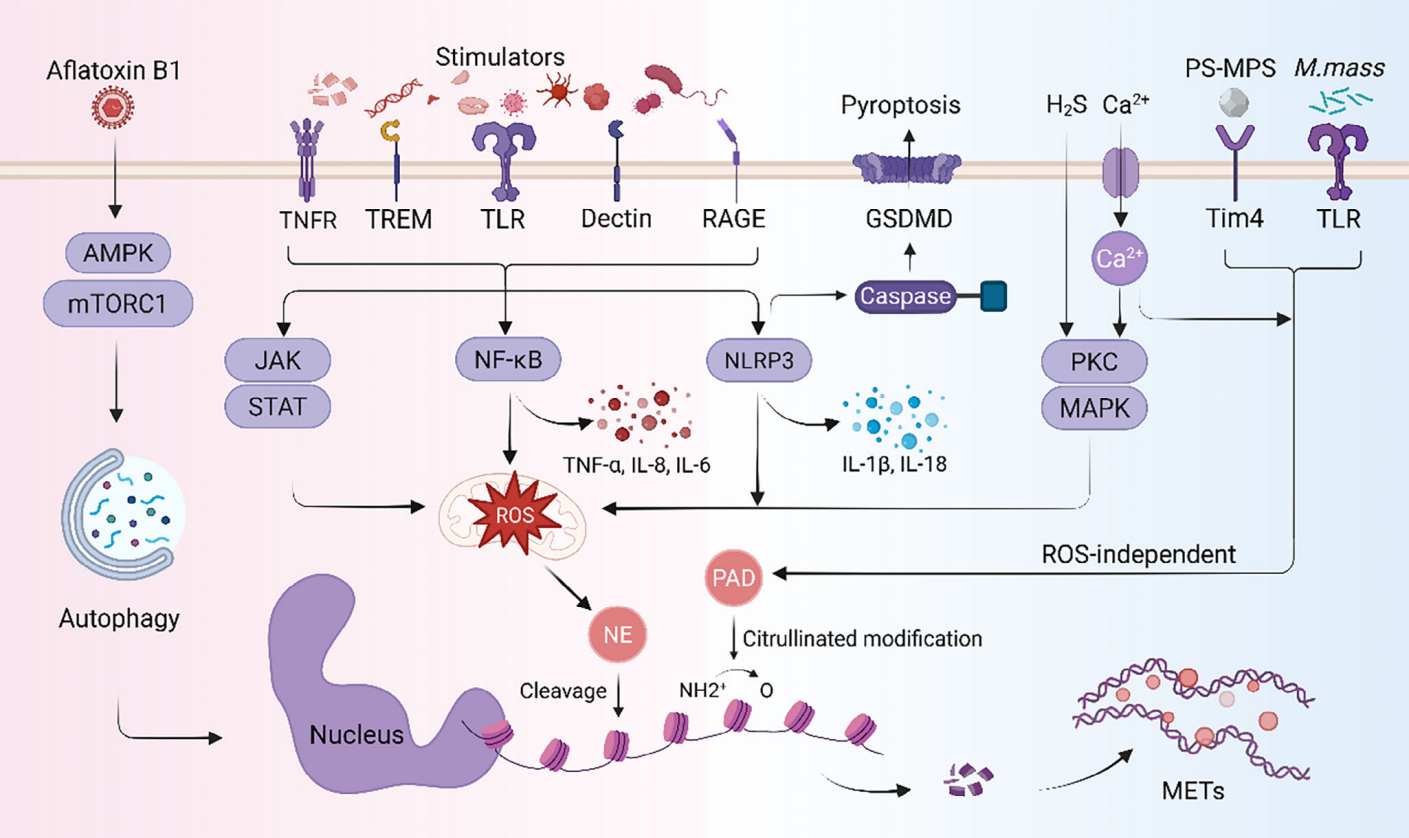
Mechanisms of METs release. After the stimulant binds to the pattern recognition receptor on the surface of macrophages, it induces the formation of METs through ROS‐dependent and ‐independent pathways. This process mainly involves the activation of multiple keys signaling pathways such as NF‐κB, NLRP3, MAPK, and STAT, which jointly promote the translocation and cleavage of NE to histones and the citrullination modification mediated by PAD, ultimately triggering the release of METs. In addition, the autophagy regulated by the AMPK pathway and the pyroptosis mediated by gasdermin also participate in the regulation of the release process of METs. Created in https://BioRender.com.

The release of METs is regulated by multiple signaling pathways, notably the NADPH/ROS pathway and PAD‐mediated histone citrullination (Figure 2). Activation of NADPH oxidase drives ROS production, thereby promoting chromatin decondensation [58, 59, 60]. Studies involving aflatoxin B1 (AFB1), kidney injury, chronic obstructive pulmonary disease, and *Streptococcus agalactiae* demonstrate that inhibiting ROS production with diphenyleneiodonium (DPI), a specific NADPH oxidase inhibitor, significantly suppresses METs formation, confirming the importance of the NADPH/ROS axis [7, 50, 61]. Concurrently, PAD, particularly PAD4 and PAD2, catalyze histone citrullination (e.g., CitH3), facilitating chromatin unpacking and METs release [62]. PAD4 deficiency reduces METosis in diabetic models, and the PAD2‐specific inhibitor PAD2‐IN‐1 suppresses METs in CC contexts [12, 63]. Notably, certain stimuli, such as *M. mass*, trigger METosis independently of NADPH/ROS through Ca^2^^+^‐dependent pathways, underscoring the mechanistic diversity in METs induction [57] (Figure 2). Therefore, whether the specific mechanisms of METosis are associated with the classical NADPH/ROS pathway requires further in‐depth investigation. Moreover, whether METosis results in cell death remains subject to debate. Under strong inflammatory stimuli (e.g., TNF‐α, HOCl, IFN‐γ), METs release is accompanied by loss of membrane integrity and cell death [26, 34]. In contrast, *Mayaro virus* (*MAYV*)‐infected human macrophages release METs without LDH leakage or membrane rupture, indicating a non‐lytic form of ETosis [64]. This discrepancy may be attributed to differences in the intracellular concentration of inflammatory mediators induced by specific stimuli [65].

Macrophages regulate the release of METs through multiple mechanisms. Their polarization states (M1 and M2) exhibit differences in the ability to release METs across various microenvironments. M1‐polarized macrophages exhibit a greater propensity for METosis, as seen in THP‐1 models where METs were predominantly released by M1‐like cells [66]. By contrast, M2‐derived METs are observed in sorafenib‐treated hepatocellular carcinoma models [67]. Interestingly, METs release and macrophage polarization form a positive feedback loop that METs further promote polarization toward the M1 phenotype, thereby exacerbating pathological processes [68]. Consequently, modulating macrophage polarization becomes an important strategy for interfering with METs formation [69]. Notably, the compositional plasticity of METs is markedly influenced by the context of their release. Stimulus‐specific variations are evident; for instance, nigericin stimulation yields METs with a mitochondrial protein content as high as 40%, compared to 10% with TNF‐α and 20% with PMA [20]. Furthermore, the macrophage polarization state dictates the protein profile of secreted METs. Pro‐inflammatory macrophages generate METs enriched in bactericidal proteins such as MPO and MMP12 [38]. This stimulus‐ and cell state‐dependent heterogeneity in composition likely provides the molecular basis for the functional divergence of METs in downstream immune responses.

Moreover, METosis is closely linked to other macrophage processes such as phagocytosis and autophagy. Sperm‐induced METs is partially dependent on phagocytosis; treatment with the phagocytosis inhibitor cytochalasin D reduces both sperm uptake by macrophages and subsequent METs release, indicating a synergistic interaction between phagocytosis and METosis [70]. Conversely, in the case of PS‐MPs‐induced METosis, larger PS‐MPs trigger significantly more METs than smaller ones, suggesting that ETosis may compensate for limitations in phagocytic capacity [71]. Nevertheless, the signals that determine the choice between phagocytosis and ETosis remain poorly characterized. Notably, autophagy also regulates METosis (Figure 2). AFB1 induces both autophagy and METs release in macrophages, and inhibition of autophagy blocks METs formation, indicating that METs release in this setting is autophagy‐dependent [61]. Thus, METs do not operate in isolation but are integrated with phagocytosis, autophagy, and polarization within a broader immune regulatory network.

## 3 METs in Immunity and Inflammation Regulation

Functioning as pivotal effector mechanisms in innate immunity, METs play a crucial role in host defense and the pathogenesis of chronic tissue damage. During the initial phase of infection or tissue injury, recalcitrant pathogens such as fungal hyphae, or environmental stimuli like hydrogen sulfide and atmospheric particulate matter, potently activate macrophages and induce METosis [72, 73, 74]. This process releases web‐like structures composed of a DNA scaffold decorated with histones and granule proteins, which effectively sequester and eliminate pathogens while rapidly initiating acute inflammatory responses. However, when the clearance of METs is impaired, or their production becomes dysregulated, this initially protective acute inflammation may transition into a chronic state, predisposing tissues to a spectrum of inflammation‐associated disorders. For instance, in models of rhabdomyolysis‐induced acute kidney injury, METs release triggered by damaged muscle exacerbates renal tubular injury [55]. Analysis of coronary artery specimens from myocardial infarction patients reveals that organized thrombi in later stages are enriched with various ETs, predominantly composed of METs [3]. Nevertheless, the mechanism by which METs shift from mediating host defense to driving pathological immune imbalance has yet to be fully elucidated. Chronic inflammation fosters a microenvironment replete with signaling molecules and reactive species, which sustains cellular proliferation, causes DNA damage, and inhibits apoptosis, thereby laying the groundwork for cancer initiation and progression [9]. Indeed, numerous malignancies, including colorectal and hepatocellular carcinomas, frequently arise against a background of persistent chronic inflammation [10, 11].

**FIGURE 3:**
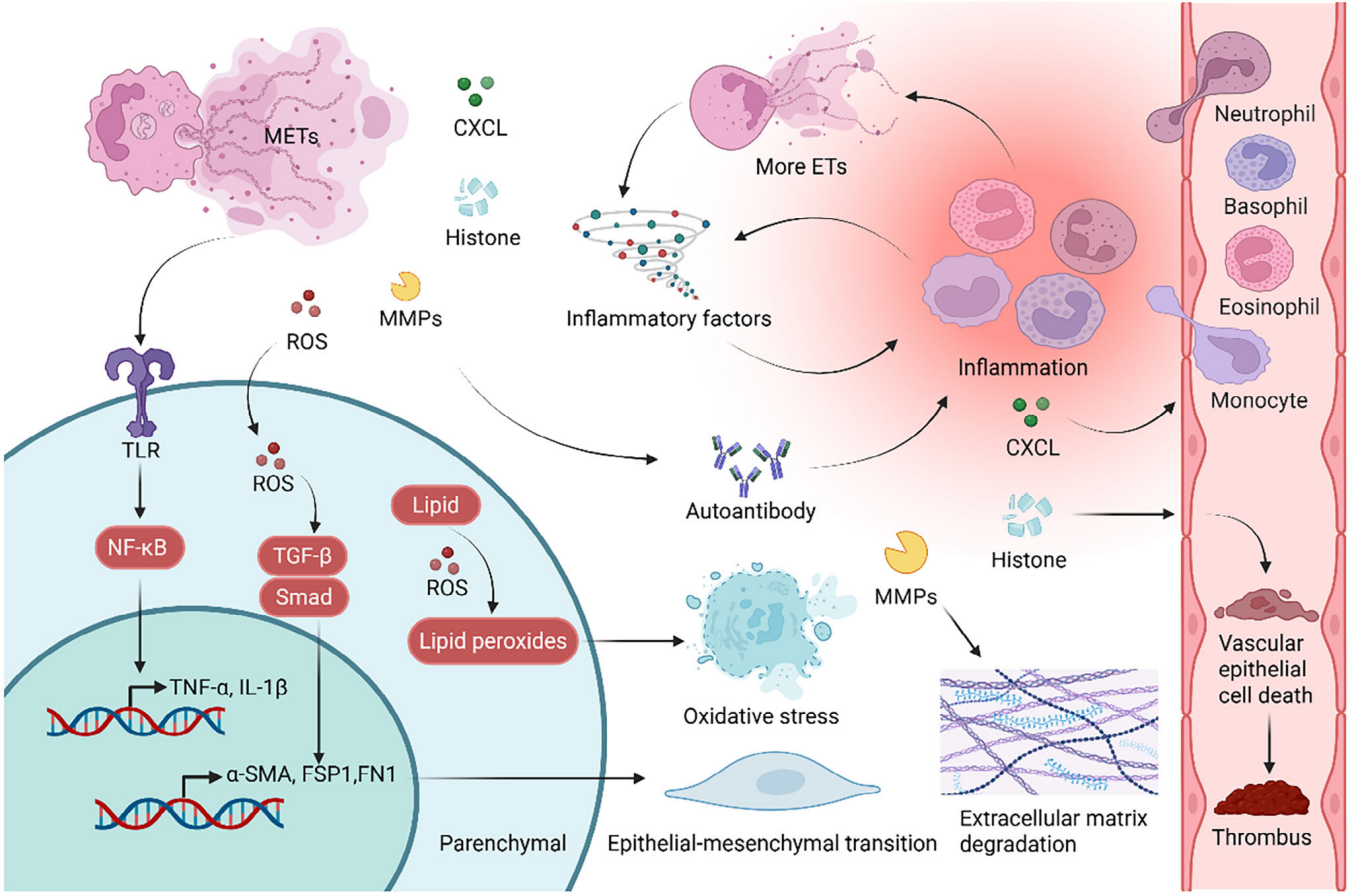
METs exacerbate tissue inflammation. The release of METs has been observed in various inflammatory conditions, including airway inflammation and neuroinflammation, where they actively contribute to the progression of inflammatory responses. As DAMPs, METs activate the NF‐κB pathway in parenchymal cells, thereby promoting inflammation. Furthermore, the excessive production of ROS during METosis not only induces oxidative stress but also activates the TGF‐β/Smad pathway, which subsequently triggers EMT. Additionally, the substantial release of free histones and effector proteins, such as MMPs, during METosis further exacerbates tissue injury. Concurrently, inflammatory cytokines released by METs, including IL‐1β and TNF‐α, recruit additional immune cells to the site of inflammation, establishing a positive feedback loop that perpetuates and intensifies the progression of inflammatory diseases. Created in https://BioRender.com.

METs drive this chronic inflammatory cascade through multiple mechanisms (Figure 3). Cell‐free DNA released from METs can be internalized by neighboring cells, activating the cGAS sensor and initiating downstream inflammatory signaling pathways. In RA, METs stimulate the DNA sensor cGAS in fibroblast‐like synoviocytes (RA‐FLS), leading to significant enrichment of genes associated with the PI3K/Akt pathway, thereby promoting RA‐FLS proliferation, invasiveness, and transition to an inflammatory phenotype [75]. Concurrently, extracellular histones released during METosis exhibit direct cytotoxicity and impair cellular metabolism and viability, potentially through TLR‐dependent mechanisms [76]. The degradation of extracellular matrix (ECM) components by various METs‐associated proteases, such as NE and MMPs, constitutes a key mechanism of tissue destruction, thereby driving the hallmark pathology of chronic obstructive pulmonary disease (COPD) and idiopathic pulmonary fibrosis (IPF) [77]. In IPF patients, METs constitute the predominant ETs subtype in bronchoalveolar lavage fluid, with their levels positively correlating with disease severity [78]. Cigarette smoke triggers METosis alongside increased expression of proteases like NE, MMP9, and MMP12, exacerbating the protease‐antiprotease imbalance and thereby driving COPD progression [79, 80]. Notably, NE itself can cleave histone H3 to activate further METs release, creating a self‐amplifying inflammatory cycle [7]. Thus, the repertoire of proteins co‐released with METs influences disease progression by remodeling the immune microenvironment, as demonstrated in spinal cord injury (SCI), where serum concentrations of METs‐associated proteins reflect injury severity [68].

Additionally, METs release modulates the expression of numerous critical factors, thereby amplifying tissue inflammation and injury. A prominent consequence is the substantial release of inflammatory cytokines. Within the SCI lesion, METs released by infiltrating macrophages promote macrophage/microglial polarization toward the pro‐inflammatory M1 phenotype via the LL37‐P2×7R‐NF‐κB signaling axis, leading to the secretion of cytokines such as IL‐1β and perpetuating the inflammatory response [68]. METs also facilitate the recruitment of other immune cells by promoting chemokine activation. In the diabetic NOD mouse model, elevated colonic METs levels enhance transcription of the pro‐inflammatory chemokine CXCL10. Blocking the CXCL10/CXCR3 signaling axis with the inhibitor AMG487 alleviates intestinal inflammation, reduces pro‐inflammatory T‐cell infiltration in peripheral tissues, and improves insulitis [63]. In the laser‐induced retinal injury model, METs promote early infiltration of neutrophils and cytotoxic T cells into the damaged photoreceptor layer, facilitating tissue repair [81]. These observations illustrate the diverse effects of METs on tissue homeostasis through modulation of distinct immune cell states. Simultaneously, METs activate epithelial transformation factors such as TGF‐β, promoting fibrotic processes. In a PS‐MPs‐induced liver fibrosis model, METs upregulated the protein expression of pro‐inflammatory genes (TNF‐α and IL‐1β) in the mouse hepatocyte cell line AML12 cells. Changes in epithelial‐mesenchymal transition (EMT) related proteins (e.g., α‐SMA, FSP1, FN1, etc.) and inflammatory factors in AML12 hepatocytes under co‐culture conditions were proportional to the release of METs, which led to an increased inflammatory response and EMT in hepatocytes through activation of the ROS/TGF‐β/Smad2/3 signaling axis [71]. It is noteworthy that tissue fibrosis significantly paves the way for early cancer cells to acquire invasive and metastatic capabilities. Patients with IPF or liver cirrhosis exhibit a substantially higher risk of developing cancer than the general population [82]. In cancer contexts, activated fibroblasts secrete abundant growth factors and signaling molecules that directly stimulate tumor cell proliferation, survival, and invasion [83]. Furthermore, high levels of cancer‐associated fibroblasts (CAFs) are often correlated with poor prognosis in various cancers [84, 85]. Therefore, by regulating the expression of key factors such as TGF‐β, TNF‐α, and CXCL, METs drive the persistence of chronic inflammation and sculpt a tumor‐promoting microenvironment, thereby establishing a solid foundation for eventual neoplastic transformation.

Moreover, METs present a vast array of self‐antigens and induce cell death, amplifying their pro‐inflammatory role. In autoimmune diseases such as RA, METosis leads to exposure of citrullinated autoantigens (e.g., citrullinated histones). These antigens, complexed with the DNA backbone of METs, form immunogenic complexes recognized by autoantibodies such as anti‐citrullinated protein antibodies, thereby triggering adaptive immune responses. These complexes also directly activate inflammatory pathways like cGAS/PI3K/Akt in fibroblast‐like synoviocytes, promoting their proliferation, invasion, and secretion of inflammatory factors [39, 86]. Importantly, ETs release pro‐inflammatory cytokines and chemokines that further recruit immune cells, which in turn release additional ETs, establishing a self‐amplifying loop [87]. Co‐incubation of macrophages with RA patient serum induces the formation of zonal METs structures, while purified METs co‐cultured with RA‐FLSs significantly promote aberrant proliferation and upregulate expression of pro‐inflammatory cytokines such as TNF‐α and IL‐1β, thereby accelerating inflammatory responses and disease progression [75]. The patients with RA exhibit a significantly elevated risk of lung, certain blood, and infection‐related cancers [88]. Considering the established contribution of METs to RA progression, a plausible mechanistic link exists whereby METs may facilitate the transformation from chronic synovial inflammation to lymphomagenesis. Additionally, the high levels of ROS released during the METosis process are closely related to immune cells death, especially ferroptosis. In models of renal and hepatic ischemia‐reperfusion injury, METs release has been identified as a key driver of ferroptosis in parenchymal cells; conversely, inhibiting METs formation significantly attenuates ferroptosis and associated tissue damage [56, 69]. Necrotic cells resulting from such damage further release DAMPs, enhancing phagocytosis and amplifying the inflammatory cascade. A fundamental question in understanding the pathogenicity of METs is how the autoantigens and cellular debris they present disrupt the homeostatic immune tolerance typically maintained by the body.

Consequently, while METs function as a key antimicrobial defense by sequestering pathogens during acute insults, they promote malignant progression in chronic disease. Research has revealed that excess NETs are cleared through macrophage uptake or by targeting neutrophil‐specific molecules like BCL‐10 to suppress NETosis [89, 90]. In contrast, the delicate balance controlling METs turnover, specifically the thresholds that dictate their generation and the immune mechanisms ensuring their removal, remains elusive. Unabated METs release stimulates a potent inflammatory signature, recruiting additional immune cells and establishing an escalating feed‐forward loop of inflammation. Critically, METs components themselves are cytotoxic, directly compromising tissue integrity, triggering cell death, and initiating pathological remodeling like fibrosis. The METs‐driven persistent inflammation creates a profoundly oncogenic environment. In this setting, abundant ROS and cytotoxic factors inflict genomic instability, while cycles of tissue damage and repair remodel the microenvironment to facilitate cancer growth and spread. This vicious cycle underscores how METs function as the cornerstone of a pathological continuum, directly connecting acute injury and chronic inflammation to cancer development.

## 4 METs in Cancer Development

A key element of evolutionary and ecological processes in cancer development and treatment is the regulation of the TME [91]. TME recruits phagocytes, mainly tumor‐associated macrophages (TAMs), to mediate the interaction between innate and adaptive immunity [92]. Plasticity and heterogeneity characteristics of TAMs mediate their tumor‐promoting and anti‐tumor effects [93]. Complex signaling networks in TME synergistically drive the pro‐cancer phenotype of immune cells and induce the release of ETs [94]. For example, mesothelin, which is secreted by pancreatic cancer cells, induces the expression of VEGF‐α (VEGF‐A) and S100A9 in macrophages. VEGF‐A feedback promotes tumor proliferation, while S100A9 drives neutrophil lung infiltration and NETs formation. This creates a vicious cycle of “tumor‐immune‐ETs” [95]. ETs are present in various processes of cancer development. Recent studies have revealed that ETs directly promote metastasis through chronic inflammation induction, ECM degradation, and the release of pro‐angiogenic factors. In addition, ETs play anti‐tumor roles by inhibiting the proliferation of tumor cells through adhesion or by enhancing the immune response [96, 97, 98]. This property may be closely related to the dynamic regulation of TME, cellular heterogeneity, and signaling pathway differences. Moreover, ETs influence the development of relevant cancers by interacting with immunoregulatory cells such as Th1 cells [99].

### 4.1 Tumor‐Promoting Effects of METs

**FIGURE 4:**
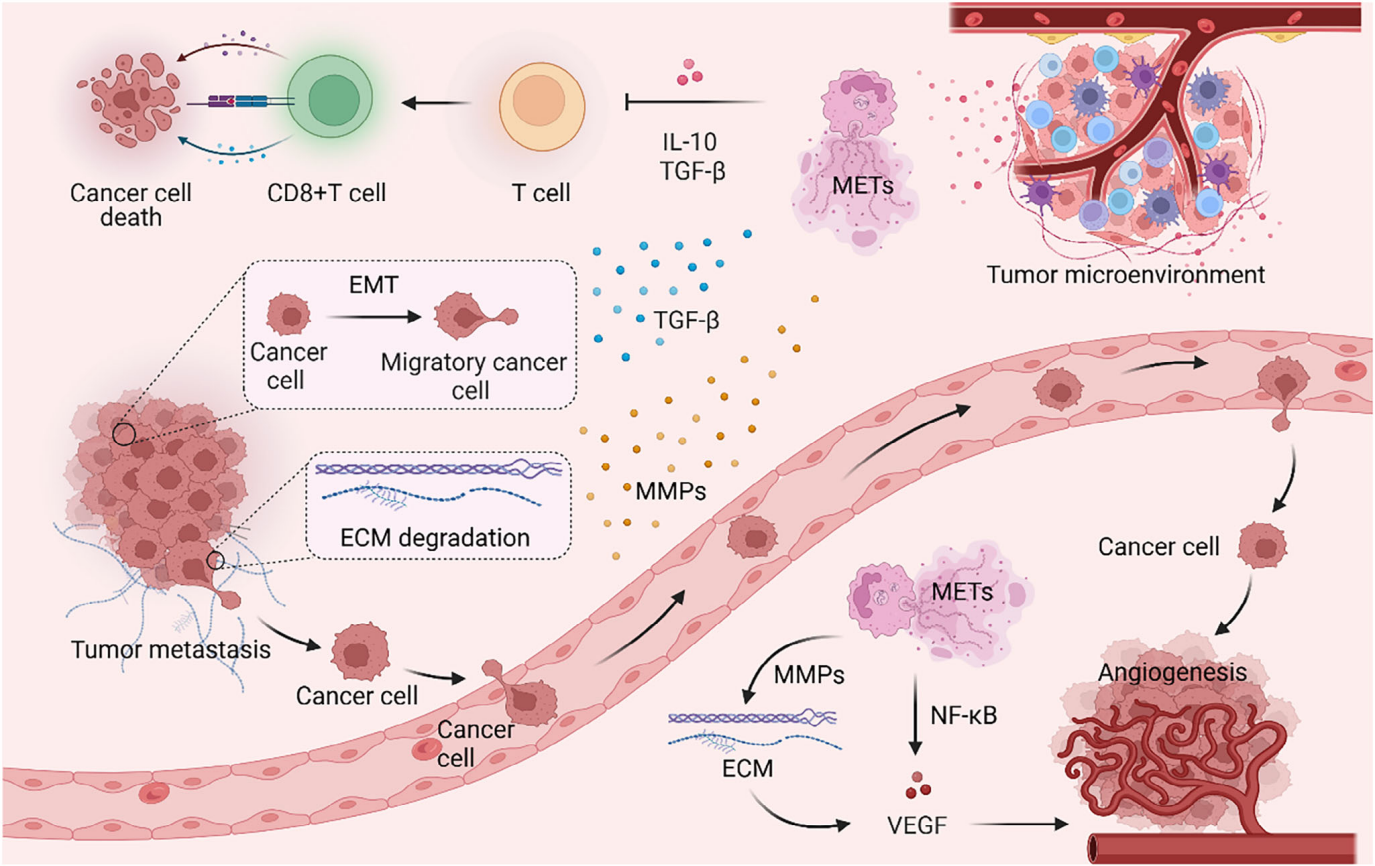
METs promote tumor development. Multiple cytokines within the tumor microenvironment stimulate TAMs to release METs. The TGF‐β/Smad pathway activated by METs induces the transformation of quiescent cancer cells into cells exhibiting a migratory and invasive phenotype, thereby enhancing their metastatic potential. METs secrete proteases, including MMPs and cathepsins, which degrade the ECM and basement membrane. This degradation creates pathways for tumor cells to invade surrounding tissues and enter blood vessels. Furthermore, METs promote angiogenesis by either upregulating the transcription and expression of VEGF through the activation of inflammatory pathways or by releasing ECM‐bound VEGF via MMPs. This angiogenesis supplies oxygen and nutrients to the tumor, supporting its rapid growth and metastasis. Additionally, factors released by METs, such as IL‐10, modulate T cell activity to induce an immunosuppressive state, thereby facilitating tumor immune evasion. Created in https://BioRender.com.

Tumor metastasis and progression arise from the coordinated interplay of multiple factors. The TME facilitates the formation of ETs, which in turn promote tumor proliferation and immune evasion through activation of pathways such as HMGB1, TLR4, and NF‐κB [100, 101, 102]. Specifically, METs contribute to tumor progression through several mechanisms. Matrix metalloproteinases (e.g., MMP9, MMP12) released during METosis degrade the ECM, while pro‐angiogenic factors such as VEGF and FGF stimulate endothelial migration and neovascularization, thereby facilitating tumor invasion and metastasis (Figure 4). In a peritoneal fibrosis model, increased METs formation coincided with upregulation of IL‐1β, TNF‐α, VEGF, and collagen I, alongside heightened vascular density and peritoneal thickening, demonstrating a correlation between METs and these pro‐tumorigenic proliferation factors [103]. Colorectal cancer (CRC) exhibits substantial macrophage infiltration within its TME [104]. METs induced by PMA enhance the invasiveness of CRC cell lines HCT‐116 and SW480. In a feedback loop, conditioned medium from HCT‐116 cells further amplifies METs release, collectively accelerating cancer progression [12]. However, the specific pathways underlying this MET‐tumor feedback cycle remain poorly defined. PDAC presents a highly immunosuppressive TME, marked by frequent metastasis and poor survival [13]. Early hepatic metastasis in PDAC is associated with elevated expression of mixed lineage kinase domain‐like pseudokinase (MLKL). MLKL‐driven necroptosis recruits and activates macrophages, which upregulate the “don't‐eat‐me” signal CD47 on tumor cells, enabling immune escape [14]. In PDAC, METs are closely linked to the CXCL8 signaling axis. Tumor‐derived CXCL8 recruits myeloid‐derived suppressor cells (MDSCs) and modulates their function [105]. Macrophages co‐cultured with MLKL‐overexpressing tumor cells release CXCL8, which promotes METs formation. METs‐derived DNA scaffolds further amplify CXCL8 production, initiating EMT and upregulating ICAM‐1 to enhance endothelial adhesion. Concurrently, METs‐associated MMPs degrade the ECM, collectively supporting PDAC liver metastasis [14].

METs also modulate anti‐tumor immunity. Sorafenib, a first‐line treatment for HCC, induces ferroptosis to suppress tumor growth [106]. However, sorafenib upregulates ARHGDIB in HCC cells, leading to IL‐4 secretion that polarizes macrophages toward an M2 phenotype and enhances PAD4 expression, thereby stimulating METs release [67]. These METs increase tumor cell viability and migration, upregulate the ferroptosis inhibitor glutathione peroxidase 4 (GPX4), and diminish sorafenib sensitivity [67]. Notably, METs exacerbate ferroptosis and aggravate parenchymal cell damage in renal injury, demonstrating an effect opposite to that observed in the tumor microenvironment [69]. METs suppress ferroptosis in cancer cells by upregulating GPX4; they conversely promote ferroptosis in normal tissues. This differential effect may stem from tissue‐specific variation in GPX4 expression. GPX4 is often overexpressed in tumor tissues compared to normal tissues and has been linked to poor prognosis in patients with hepatocellular carcinoma [107, 108]. Ferroptosis is widely regarded as a promising target in cancer therapy; however, the interaction between METs and ferroptosis within the TME remains poorly understood [109, 110]. Clarifying the role of ferroptosis in METs‐mediated immunoregulatory pathways is critical for overcoming drug resistance and developing novel strategies. Furthermore, T‐cell regulation is crucial in anti‐tumor immunity. In HCC, TGF‐β1‐induced NETs bind TMCO6 on CD8^+^ T cells, suppressing T cell receptor signaling and NF‐κB p65 nuclear translocation, thereby impairing cytotoxic responses and accelerating tumor progression [111]. Although the direct interaction of METs with T cells in tumor progression has not been clarified, a laser‐induced retinal injury model reveals that METs regulate T cell and granulocyte migration, suggesting their potential to modulate T cell function within the TME [81].

Therefore, METs facilitate tumor progression through diverse mechanisms—angiogenesis, immune suppression, ECM remodeling, and metastasis—whose output is shaped by the dynamic TME. Current evidence remains fragmented across tumor types and mechanisms. Future studies should systematically dissect METs‐related pathways in various cancers and develop targeted combinatorial approaches for therapeutic intervention.

### 4.2 Tumor Suppressive Effect of METs

Although the inhibitory role of METs in tumorigenesis and progression has not been fully established, accumulating multidimensional evidence suggests their potential involvement in modulating tumor behavior [15]. A retrospective analysis of 15 glioblastoma patients revealed that the two individuals exhibiting METs formation had the most favorable prognosis, with survival durations of 3.2 and 4.6 years, respectively, whereas non‐METs patients survived between two months and 2.8 years [15]. Patients who developed METs survived longer than patients with glioblastoma who did not develop METs [15]. This correlation implies that METs may contribute to restraining aggressive tumor growth through specific molecular pathways.

As pivotal effector cells within the TME, TAMs influence tumor behavior through multiple mechanisms, including phagocytosis of tumor cells and subsequent activation of adaptive immunity [112]. For example, in pancreatic cancer, miR‐340 downregulates CD47 expression, enhancing macrophage‐mediated phagocytosis and inhibiting tumor growth [113]. METosis is significantly associated with phagocytosis. In response to external stimuli, METs occur simultaneously with phagocytosis, acting as an effective complement to it [71]. Therefore, METs may be extruded as part of the tumor‐suppressive response of macrophages, extending their phagocytic function. Furthermore, M1‐polarized macrophages potently inhibit tumor growth by releasing effector molecules such as ROS and HMGB1, and their phagocytosis of tumor cells initiates systemic T cell‐mediated immunity [114, 115]. Given that METs are more frequently released by M1 macrophages, they may exert anti‐tumor effects through the direct cytotoxicity of associated proteins and by impeding metastatic dissemination.

Research on the tumor‐inhibitory mechanisms of METs remains nascent, whereas the role of NETs in tumor suppression is better characterized. For instance, neutrophil elastase and other proteases associated with NETs induce apoptosis in CRC cells by promoting mitochondrial translocation of BAX [116]. Additionally, NETs‐derived DNA scaffolds can bind melanoma cells via integrin‐mediated adhesion, suppressing their migration and viability [117]. In this study, NETs were equally found to be cytotoxic to melanoma cells in vitro [117]. These findings indicate that ETs can inhibit tumor proliferation through cytotoxic effects and physical containment. Notably, METs and NETs share structural homology—both consist of a DNA backbone decorated with proteins such as MPO, NE, and MMPs [20, 50, 118, 119]. This suggests that METs may similarly impede tumor progression via protease‐mediated cytotoxicity and DNA‐based physical barriers. Lactoferrin has been detected as immunopositive within METs structures in glioblastoma [15]. Notably, this protein has been established in prior studies to possess anti‐tumor activity, such as inducing tumor cell cycle arrest [120, 121]. Moreover, lactoferrin promotes macrophage polarization toward the M1 phenotype, which is associated with anti‐tumor functions [122]. Therefore, core protein components of METs may harbor intrinsic potential to directly eliminate tumor cells.

Thus, clinical prognostic data and mechanistic analogies with NETs support the hypothesis that METs may suppress tumor development. METs could potentially inhibit tumors by releasing cytotoxic proteases, physically trapping malignant cells, and synergizing with phagocytosis to enhance immune clearance. Nevertheless, research on the tumor‐suppressive functions of METs remains relatively scarce, with many mechanistic insights and functional relevance still poorly characterized. Despite these gaps, the multifaceted biological effects of METs already demonstrate considerable anti‐tumor potential. Future studies should systematically dissect the dynamic regulatory networks governing METs within the TME to clarify their context‐dependent functions in oncology.

### 4.3 Dual Prognostic Role of METs in Cancer Progression

Assessing disease prognosis is a key part of making clinical treatment decisions. Growing evidence suggests that ETs may serve as novel tumor prognostic markers [123]. Patients with elevated MPO‐DNA levels in tumor tissue and serum have a poorer prognosis. For instance, in cases of advanced gastric cancer or diffuse large B‐cell lymphoma, NETs contribute to increased risks of proliferation and metastasis, correlating with reduced overall survival [123, 124]. Similarly, METs appear to hold prognostic value. Histological analysis of 116 CRC patients revealed significantly higher METs levels within the tumor core compared to adjacent normal tissues [12]. High METs expression was strongly associated with distant metastasis, and cancer‐specific mortality was 2.18‐fold greater in the high‐METs group, indicating a negative prognostic impact [12]. In a study of 135 patients who underwent radical resection for nonfunctional pancreatic neuroendocrine tumors, those with high infiltration of neutrophils or macrophages, particularly those expressing METs or NETs, exhibited significantly worse recurrence‐free survival [125]. These findings collectively underscore the association between METosis and cancer progression, supporting the potential use of METs as a prognostic biomarker.

However, a paradoxical pattern emerges in glioblastoma. Pathological evaluation of 15 patients revealed that METs derived from M2 macrophages, localized at the tumor–necrosis interface, were correlated with a favorable prognosis. Patients with detectable METs survived significantly longer than those without [15]. METs may act as a positive prognostic marker by suppressing tumor invasion. It is important to note that the evidence linking METs to a favorable prognosis in glioblastoma is derived primarily from studies with limited patient cohorts. These findings remain preliminary, and the underlying biological mechanisms have yet to be elucidated. A similar beneficial association has been reported for NETs in high‐grade serous ovarian carcinoma, where their presence correlated with improved overall survival, elevated S100A8 expression, and increased regulatory T cells in milky spots [126]. These observations imply that the favorable prognostic effect of ETs may stem from their ability to remodel the immune landscape of the TME. Notably, while the TME regulates METs release, METs themselves can alter the TME through the secretion of chemokines such as CXCL8 [14]. Therefore, future studies should focus on dynamic TME features, such as shifts in immune cell composition and protein expression profiles, as a means to elucidate the precise mechanisms by which METs influence tumor progression and patient prognosis.

### 4.4 Are METs Friends or Foes during Cancer Progression?

**FIGURE 5:**
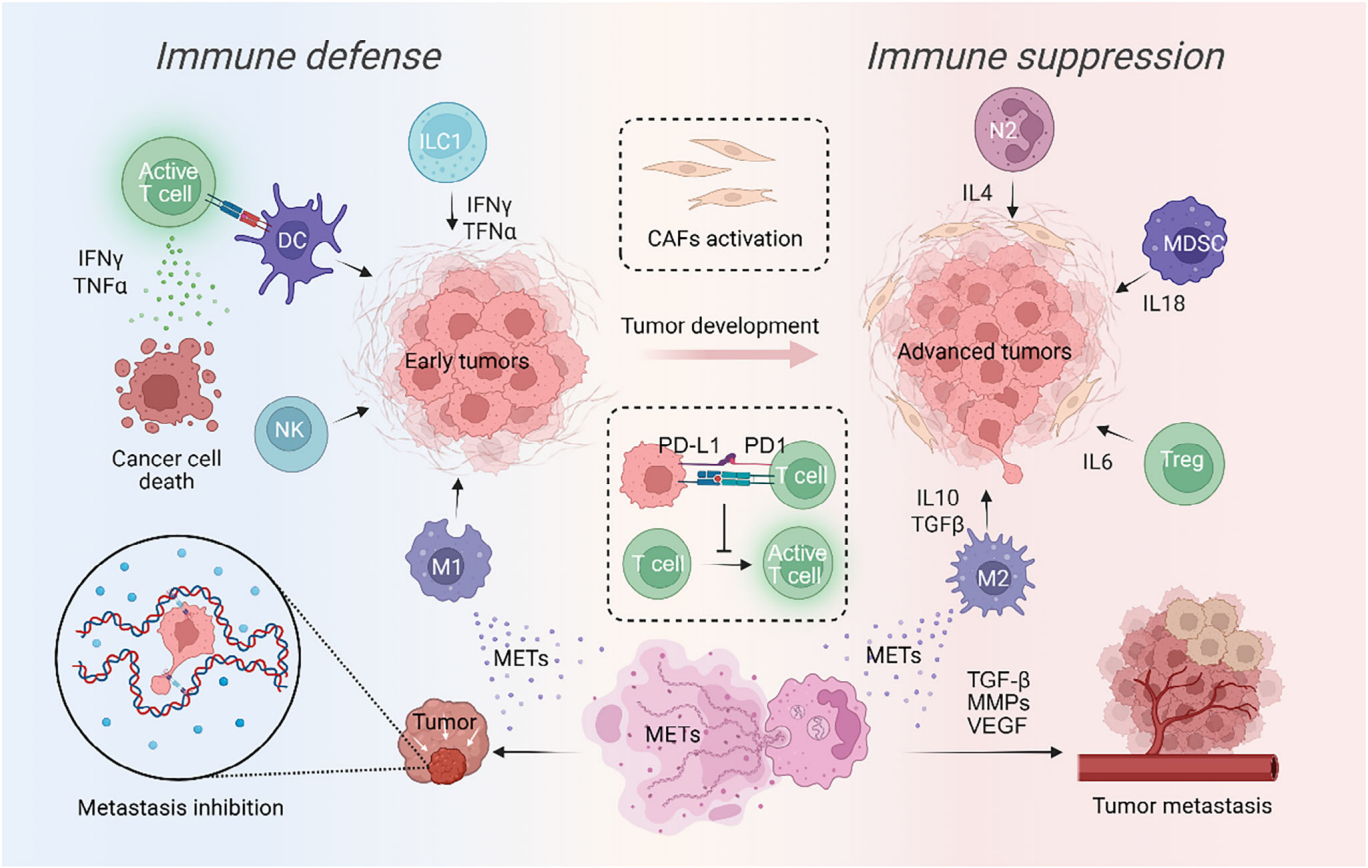
The dynamic changes of the TME determine the expression of pro‐tumorigenic or anti‐tumorigenic functions of METs. In the early stages of tumor development, the tumor immune microenvironment is highly immunogenic. Dominated by NK cells and effector T cells, it initiates anti‐tumor immune responses, mediating cancer cell killing through the secretion of factors like IFN‐γ and TNF‐α. During this phase, METs are induced to perform anti‐tumorigenic functions, adhering to cancer cells to suppress metastasis. As the tumor progresses, inhibitory signaling pathway become activated. CAFs secrete abundant collagen and hyaluronic acid, remodeling the extracellular matrix and forming a dense physical barrier. Concurrently, Treg cells and MDSCs (myeloid‐derived suppressor cells) undergo significant expansion, and M2‐polarized TAMs emerge, collectively establishing a potent immunosuppressive network. Within this altered microenvironment, METs shift their function, upregulating the secretion of factors such as TGF‐β, MMPs, and VEGF to promote tumor metastasis. Created in https://BioRender.com.

The paradoxical prognostic significance of METs underscores their context‐dependent ability to either promote or suppress tumor progression. In the tumor‐promoting role, METs enable tumor invasion and metastasis by degrading the ECM, facilitating EMT, and promote angiogenesis [12, 14, 67]. Conversely, METs exhibit tumor‐suppressive potential by inducing apoptosis through protease release, inhibiting proliferation via adhesion interference, and enhancing immune‐mediated clearance through phagocytosis cooperation [15, 117]. The molecular switch governing these opposing functions likely depends on the stage of tumor progression and the degree of heterogeneity within the TME (Figure 5).

A key determinant of METs’ functional orientation appears to be the immune context of the TME. An immunogenic TME is more likely to direct METs' effects toward anti‐tumor immune activation, whereas an immunosuppressive TME tends to exploit METs to promote tumor progression. During early tumorigenesis, the TME is typically dominated by effector T cells and NK cells, exhibiting strong immunogenicity. Recruitment of M1‐polarized TAMs facilitates tumor cell proliferation suppression [127, 128]. ETs released during this phase tend toward anti‐tumor expression. For example, in pancreatic cancer, the recruitment and polarization of M1‐like macrophages and increased CD8^+^ T cell levels enhance the anti‐tumor effect of miR‐340 achieved through promoting macrophage phagocytosis [113]. Within such an immunogenic setting, METs may act synergistically with macrophage phagocytosis by physically trapping tumor cells and inhibiting dissemination. Similarly, NETs capture circulating tumor cells and suppress invasion through β1‐integrin‐mediated adhesion of early cancer cells, further demonstrating how early TMEs promote anti‐tumor ETs properties [129]. Thus, an immunogenic TME early in tumor development favors anti‐tumor METs activity.

As tumors progress toward uncontrolled growth, the TME shifts toward immunosuppression, characterized by increased levels of IL‐10, ROS, and iNOS. Treg cells and MDSCs expand, while TAMs repolarize to an M2‐like state, establishing an immunosuppressive microenvironment [127, 128, 130]. MMT‐derived CAFs from M2 TAMs significantly promote tumor metastasis [131]. In this setting, METs often adopt pro‐tumorigenic functions. In PDAC, METs contribute to an immunosuppressive milieu rich in CXCL8, thereby promoting metastasis [14]. In triple‐negative breast cancer, hyperactivated Stat3 fosters a suppressive environment that limits CD8^+^ T cell activity, wherein ETs facilitate tumor progression [99]. Similarly, in a mouse model of colorectal liver metastasis, TAM‐derived extracellular vesicles establish a pro‐invasive niche that enhances NETs formation, which in turn accelerates tumor growth [132]. Therefore, strongly immunosuppressive TMEs may drive METs toward pro‐tumorigenic expression. Moreover, factors co‐released with ETs, such as TGF‐β, further reinforce immunosuppression, particularly through T cell modulation. Multiple studies indicate that ETs carrying PD‐L1 inhibit T cell function, and suppression of ETs restores T cell activity and reduces metastasis [133]. This positive feedback between ETs and immunosuppression markedly accelerates tumor progression [134].

During tumor development, the reprogrammed TME significantly suppresses local residual and distant tumors while inducing strong immunological memory effects [135]. Thus, reprogramming the immunosuppressive TME has emerged as a promising therapeutic strategy. For example, EV‐based nanodrugs inhibit TGF‐β, leading to significant activation of CD8^+^ T cells and an increased M1/M2 macrophage ratio, suppressing glioblastoma growth and extending survival in mouse models [136]. Calceolarioside B binds and inhibits MMP12 in macrophages and liver cancer cells, reducing M2‐like TAM polarization and infiltration, thereby alleviating immunosuppression and inhibiting hepatocellular carcinoma progression [137]. Notably, blocking ETs' release effectively improves the immunosuppressive TME [138]. In breast cancer mouse models, PAD4 inhibitors significantly reduce ETs formation and suppress tumor growth in a concentration‐dependent manner, manifesting potent anti‐tumor properties [139].

The inflammatory signaling network within the tumor microenvironment dynamically orchestrates the release and functional polarization of METs. Key pathways, including NF‐κB, NLRP3, MAPK, and JAK‐STAT, are central to the process of METosis and collectively shape tumor progression by regulating mechanisms such as the cell cycle and immune responses. Inflammatory cytokines such as TNF‐α, IL‐1β, and IL‐6 act as central hubs, differentially activating downstream signaling to steer METs toward either pro‐tumor or anti‐tumor phenotypes. Under anti‐tumor conditions, acute, high‐intensity alarm signals (e.g., strong TNF‐α, mature IL‐1β) activate a distinct program involving NF‐κB, NLRP3 inflammasome priming, exerting anti‐tumor effects through mechanisms such as inducing cell pyroptosis [140, 141]. This drives macrophages toward an M1 state and triggers the release of METs enriched in anti‐tumor effectors such as MPO and MMPs, directly inhibiting tumors. Although METs release may be lower in this setting, its components show greater anti‐tumor specificity and cause less systemic immune disruption. In contrast, chronic, low‐grade signals (e.g., TNF‐α, IL‐6) sustain activation of NF‐κB, JAK‐STAT3, and ERK‐MAPK, promoting an immunosuppressive microenvironment and M2‐like macrophage polarization [142, 143, 144, 145]. This leads to high MET levels, which further enhance immunosuppression and angiogenesis via factors like TGF‐β and VEGF, creating a self‐reinforcing loop that accelerates tumor growth.

Therefore, the role of METs in tumor progression is dynamically shaped by the immune status of the TME. In early, immunogenic microenvironments, METs tend to exert anti‐tumor effects, whereas in advanced cancers within immunosuppressive TMEs featuring elevated inhibitory factors like CXCL8, METs shift toward pro‐tumorigenic functions. However, many current studies rely heavily on in vitro systems or correlative observations, without adequate in vivo validation, as well as mechanistic insights into the regulation of METs' functions in tumor promotion and suppression remain scarce. In addition, research on NETs and other extracellular trap populations may confuse the interpretation. Present hypotheses regarding how the METs' functional phenotype is modulated are inferred primarily by analogy to the more extensively studied behavior of NETs. Future studies should clarify the precise contribution of ETs to the establishment and maintenance of immunosuppressive niches and explore ETs‐targeted strategies to reverse immune evasion and reinstate anti‐tumor immunity.

## 5 METs in Cancer Immunotherapy

Despite the widespread use of conventional cancer treatments such as chemotherapy and radiotherapy, they often inflict significant collateral damage. Cancer immunotherapy offers a more targeted approach by mobilizing the body's immune system against tumors. Its effectiveness, while promising, is limited by the tumor's ability to evade immune detection [16]. Within the tumor microenvironment, the release of METs promotes tumor metastasis by inducing angiogenesis and suppressing immune responses [14, 103]. It may also amplify inflammatory reactions, which could paradoxically activate anti‐tumor immunity [15]. Therefore, understanding how METs influence the efficacy of cancer immunotherapy remains a critical question in this field.

### 5.1 How METs Affect Cancer Immunotherapy

During tumor progression, cancer cells evade immune surveillance through mechanisms such as upregulating immune checkpoint molecules and recruiting immunosuppressive cells. Conventional chemotherapy can inhibit tumor growth by targeting rapidly dividing cells, but its lack of specificity often damages the immune system [146]. In contrast, cancer immunotherapy is designed to harness and enhance the patient's own immune system to precisely recognize and eliminate tumor cells. By improving antigen recognition and cytotoxic function, immunotherapy offers a better safety profile and can establish long‐term immune memory, reducing risks of recurrence and metastasis [16]. Currently, several immunotherapeutic approaches have shown remarkable clinical efficacy, including immune checkpoint inhibitors (e.g., PD‐1/PD‐L1 antibodies, CTLA‐4 antibodies), CAR‐T cell therapy, and cancer vaccines. Nonetheless, immunotherapy still confronts challenges, including significant individual variability in response, drug resistance, and the heterogeneity of the tumor immune microenvironment [18]. Owing to their significant yet paradoxical functions in oncology, METs can potentially modulate the outcomes of cancer immunotherapy in complex and context‐dependent ways.

**FIGURE 6:**
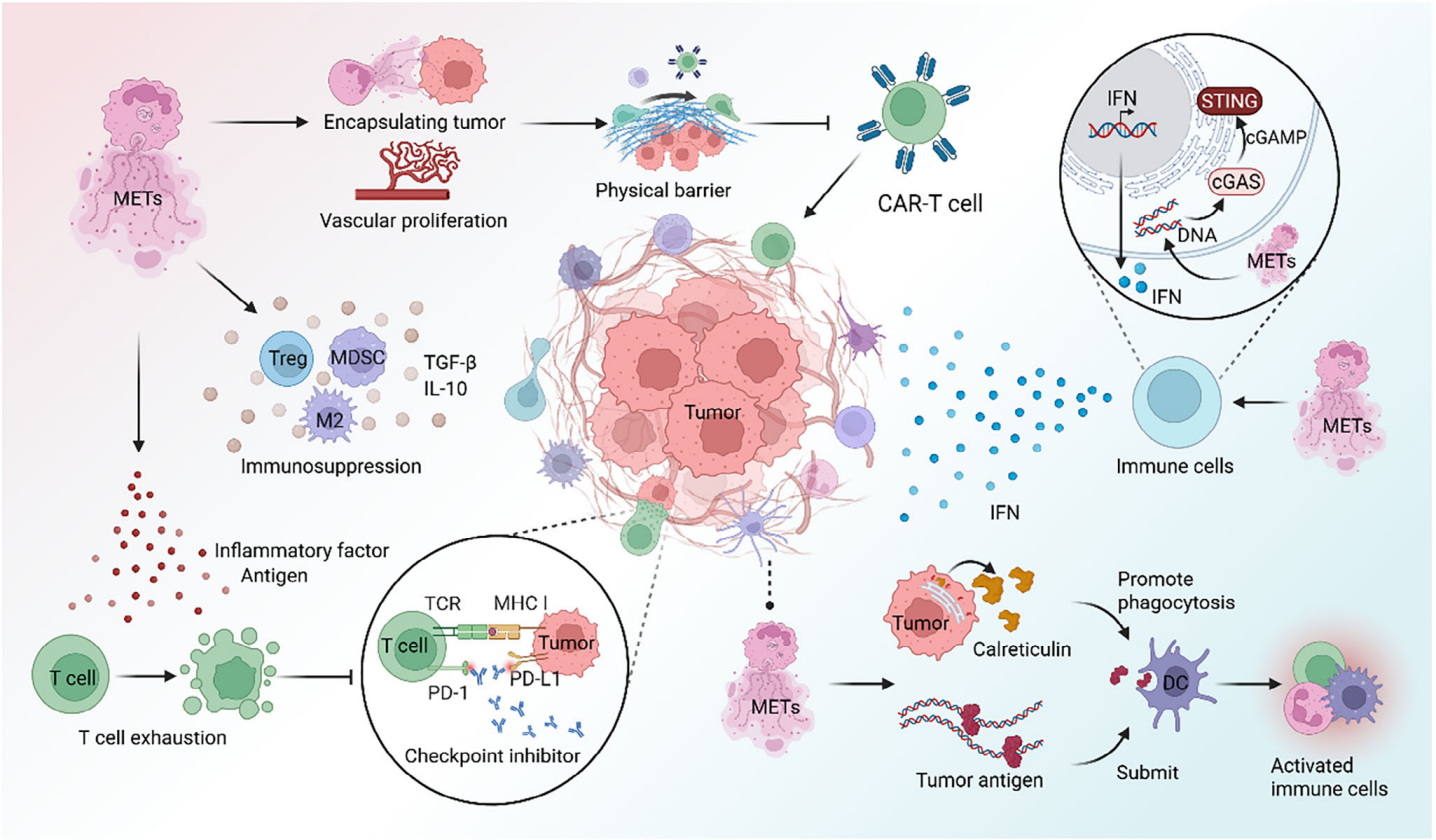
The role of METs in cancer immunotherapy. METs mediate regulatory effects on cancer immunotherapy response through dynamic TME alterations. METs entrap tumor cells and promote angiogenesis, forming a physical barrier that impedes the infiltration of immune cells (e.g., CAR‐T cells) and therapeutic agents into the tumor site. The persistent inflammatory signals and immunosuppressive factors released alongside METs induce T cell exhaustion and foster an immunosuppressive microenvironment, thereby attenuating the response to immunotherapy. Conversely, METs act synergistically to enhance cancer immunotherapeutic outcomes. The chromatin released during METosis can be internalized by immune cells, activating the cGAS‐STING pathway and promoting IFN production, which amplifies innate immune responses. Additionally, METs may serve as scaffolds for tumor antigen presentation and induce endoplasmic reticulum stress leading to CALR overexpression, thereby promoting dendritic cell recognition and phagocytosis of tumor cells. These mechanisms ultimately enhance the systemic immune activation elicited by cancer immunotherapies such as oncolytic viruses. Created in https://BioRender.com.

The pro‐tumor functions of METs may compromise the effectiveness of cancer immunotherapy (Figure 6). METs are released by macrophages in response to various stimuli and can entrap pathogens or tumor cells, thereby limiting their spread. NETs have been observed to adhere to tumor cells via integrins, mediating limited cytotoxicity [117]. Analogous to NETs, METs may adhere to tumor cells and facilitate a certain degree of cell killing. It is worth noting that METs released under specific stimulation (e.g., by *M. mass*, IFN‐γ) trap pathogens without killing them; instead, they form a barrier that concentrates pathogens, thereby promoting their growth [27, 57]. METs could also form a physical barrier within the tumor locale, impeding direct contact between effector immune cells like T cells and NK cells and their tumor targets, thereby attenuating cytotoxic capacity. Moreover, the release of various factors during ETosis, including ECM‐degrading MMPs and pro‐angiogenic factors (VEGF, FGF, which induce angiogenesis), collectively remodels the tumor microenvironment. This process provides a structural basis for tumor invasion and metastasis, resembling the vascular co‐option phenomenon commonly seen in solid tumors and the ECM remodeling mediated by CAFs [147]. Such mechanisms impede the infiltration of therapeutic immune cells, including CAR‐T cells, into tumor sites, limiting their anti‐tumor functions. Currently, the development of targeted delivery systems, such as nanoparticle‐based delivery technologies, represents a key breakthrough for enhancing therapeutic efficacy [148, 149]. Additionally, excessive METs foster an immunosuppressive microenvironment, which may reverse the anti‐tumor immunity restored by immunotherapy. The high heterogeneity and dynamic nature of the TME constitute a major challenge for current immunotherapies [150]. In advanced tumor stages, pro‐tumoral M2 macrophages predominate. The concurrent ETosis process and the associated release of immunosuppressive factors such as TGF‐β and IL‐10 promote the proliferation of Tregs and MDSCs, establishing an immunosuppressive tumor microenvironment that acts to restrict the infiltration and activation of CAR‐T cells and endogenous anti‐tumor immune cells, thereby hampering therapeutic efficacy [151, 152, 153]. It is also noteworthy that CAR‐T cell therapy can trigger a substantial systemic increase in cytokines, leading to cytokine release syndrome (CRS). Given that multiple factors released in METosis could aggravate such adverse effects. In preclinical models, employing the antibody lenzilumab to inhibit macrophage activation and GM‐CSF signaling effectively mitigated CAR‐T therapy‐associated toxicity [154, 155]. However, whether this strategy can effectively suppress METs release while inhibiting macrophage activation, and further ameliorate the immune microenvironment to enhance therapeutic outcome, remains to be thoroughly explored. Moreover, during tumor progression, METs release presents a plethora of self‐antigens, inflammatory mediators, and inhibitory factors. Persistently present antigens and inflammatory signals can gradually induce functional exhaustion in CD8^+^ T cells [156]. Early studies indicate that the extent of antigen‐specific T cell exhaustion is closely associated with antigen abundance, as well as to chronic systemic inflammation [157, 158]. Consequently, METs release may participate in driving T cell exhaustion during tumor progression. Exhausted T cells are typically characterized by progressive loss of effector functions, co‐expression of inhibitory receptors (e.g., PD‐1, LAG3), metabolic dysregulation, and impaired memory recall responses [156, 157]. The efficacy of diverse immunotherapies is directly contingent upon the quality of effector CD8^+^ T cells in the TME. This METs‐mediated T cell exhaustion compromises the capacity of T cells to effectively mediate tumor clearance, thereby facilitating tumor cell immune escape and diminishing the effectiveness of immunotherapies, including checkpoint inhibitors, that rely on T cell activation.

Nevertheless, under certain conditions, METs may act in concert with immunotherapy to exert anti‐tumor effects (Figure 6). In *MAYV*‐infection, when the virus binds extracellular DNA, METs act as a scaffold that conversely promotes *MAYV* spread to adjacent cells [64]. In oncology, METs might function as a scaffold for antigen presentation, thereby strengthening anti‐tumor immunity. Effective capture and presentation of tumor antigens by dendritic cells (DCs) is a critical step in initiating adaptive immune responses. Following administration of cancer vaccines or immune checkpoint inhibitors, highly organized macrophage clusters can cooperatively phagocytose cancer cells to suppress tumor growth [159]. Within this cooperative setting, METs may function as intercellular nets that adhere to tumor debris, thereby enhancing antigen capture and presentation by macrophages and ultimately strengthening the adaptive immune response. But be aware of whether tumor cells might also use this scaffold to aid in metastasis and escape. Moreover, ROS and inflammatory factors accumulated during METosis induce endoplasmic reticulum stress in tumor cells, leading to upregulation of surface calreticulin (CALR) [160]. Surface‐exposed CALR has been shown to promote the uptake of apoptotic tumor cells by DC precursors and effectively activate NK cells [161, 162]. A recently designed pro‐phagocytic polymeric nanocomposite promotes macrophage phagocytosis of tumor cells by coordinately upregulating the pro‐phagocytic CALR signal while suppressing the anti‐phagocytic CD47 signal [163]. Therefore, CALR upregulation induced by METs release may enhance the antigen‐uptake capacity of DCs and the cytotoxic function of NK cells. However, the molecular switches that determine whether METs restrict or facilitate intercellular transfer remain unclear.

Currently, various therapies such as oncolytic virus therapy (using genetically modified viruses to infect tumor cells) enhance systemic anti‐tumor immunity by stimulating a pro‐inflammatory tumor microenvironment [164]. In this scenario, METs potentially act as “immune amplifiers”, intensifying inflammatory signals and thereby boosting the anti‐tumor immune response. METs release is typically accompanied by abundant secretion of inflammatory factors, and the extracellular DNA released is a potent activator of the STING pathway. The cGAS–STING pathway is a key DNA‐sensing mechanism in innate immunity and antiviral defense, capable of recognizing diverse cytosolic DNA species, including mitochondrial DNA and tumor‐derived genomic instability fragments, thereby initiating immune responses [165]. Upon DNA sensing, cGAS activates STING, triggering the TBK1–IRF3 axis and NF‐κB signaling, which promote type I interferon (IFN) production in endothelial cells to delay tumor progression [165, 166]. Released METs serve as a potent ligand for the DNA sensor cGAS in fibroblast‐like synoviocytes, leading to the subsequent induction of downstream signaling [75]. Studies show that alveolar epithelial cells activate the cGAS–STING pathway by endocytosing NETs, suggesting that free DNA released during METosis may similarly induce type I interferon production via this pathway, exerting anti‐tumor effects [167]. Current research is exploring targeted activation of cGAS–STING using precision drug delivery systems; combining such strategies with anti‐PD‐L1 immunotherapy has shown significant tumor suppression in preclinical models [168]. However, the sustained activation of the cGAS–STING pathway under chronic stimulation promotes tumor growth by perpetuating inflammation and inducing an immunosuppressive TME [169]. Excessive METs release leading to STING overactivation may disrupt immune homeostasis and compromise therapeutic outcomes. Therefore, fine‐tuning METs levels to maximize synergy with other immunotherapies represents a promising avenue for future research.

Thus, while cancer immunotherapy has revolutionized patient care through its targeted approach and improved safety, its success is limited by factors such as tumor microenvironment heterogeneity. METs, through their release of diverse cytokines and proteins, function as master regulators with context‐dependent anti‐tumor and pro‐tumor activities. This functional duality necessitates a sophisticated understanding of their role. Therefore, strategically modulating METs presents a promising avenue for augmenting immunotherapy, positioning them as a compelling therapeutic target for future investigation.

### 5.2 Targeting METs in Cancer Immunotherapy

Defining the presence, distribution, quantity, and molecular composition of METs is essential for elucidating their pathophysiological roles, evaluating their biomarker potential, and designing targeted therapeutic strategies. Current detection approaches often rely on the colocalization of extracellular trap markers with macrophage‐specific identifiers [71, 170]. However, accurate discrimination of METs remains challenging due to their structural and compositional overlap with NETs—including shared components such as DNA, histones, and granule proteins—as well as the phenotypic diversity of macrophages themselves [171, 172, 173]. Given their emerging role in the pathogenesis of inflammatory diseases and cancer, METs represent a promising target for novel interventions. A major future challenge will be the development of strategies that selectively eliminate or inhibit pathogenic METs while preserving their beneficial antimicrobial functions and minimizing impact on homeostatic macrophage activities [174].

#### 5.2.1 Biomarkers of METs

Based on the structural hallmark of ETs, a DNA scaffold decorated with associated proteins, the most widely used detection method involves assessing the co‐localization of extracellular DNA with characteristic protein markers (Table 2). Since DNA forms the core structure of METs, fluorescent DNA‐intercalating dyes such as SYTOX Green, PicoGreen, and TO‐PRO are commonly employed for visualization [12, 20, 57, 175]. To improve specificity, DNase treatment is often applied prior to quantification to eliminate background DNA from non‐ET sources. Additionally, measuring ROS production can serve as a functional correlate of METs formation.

**TABLE 2:** A summary of the potential targets for the detection of METs.

Stimulus type	Cell model	Time	Targets for detection	References
Perfluorooctane sulphonate (PFOS)	Raw264.7 cells, murine liver macrophages	24 h	DNA, PAD4, MPO, H3	[36]
Polystyrene microplastics (PS‐MPs)	Chicken liver macrophages, PMA stimulates macrophages induced by peripheral blood monocytes	12 h	DNA, MPO, NE, F4/80 (marker of macrophage)	[37]
Neutrophil elastase (NE)	Murine BAL Macrophages, human blood monocyte derived macrophages (hBMDM)	2 h	DNA and CitH3	[33]
HOCl	Human monocyte‐derived macrophages (HMDM)	15 min	DNA, H3	[26]
Cigarette smoke	Alveolar macrophages, murine BAL macrophages	1 h, 4 d	DNA, CitH3, MMP9, MMP12, PAD2	[79]
*Uropathogenic Escherichia coli* (*UPEC*)	Suburothelial perivascular macrophages (suPVMs)	6 h	DNA and CitH3	[53]
*Mayaro virus* (*MAYV*)	Raw264.7 cells	9 h	DNA and CitH3, MPO, NE	[64]
Calcium oxalate (CaOx) crystals	RAW264.7 cells, CaOx mice model	48 h	DNA and CitH3, MMP12, F4/80	[31]
Platelet (injected with glycerol)	THP‐1 differentiated macrophages, C57BL6/J mice	8 h	DNA and CitH3, F4/80	[55]
Spinal cord injury (SCI)	Rats spinal cord injury (SCI) model	7 days after the operation	CitH3, CD68	[68]
Hypoxia/reoxygenation (H/R)	RAW264.7 cells	Hypoxia for 9 h and reoxygenation for 2 h	DNA, CitH3, MPO, NE, F4/80 M1 markers (CD86/iNOS), M2 markers (Arg‐1/IL‐10) dsDNA, MPO and NE for METs quantification	[69]
Rheumatoid arthritis (RA)	SF samples were obtained from inflamed knees of ACPA+ patients		DNA, CitH3, CD68, PAD4	[86]
Type 1 Diabetes	15‐week‐old NOD mice with T1D	15‐week‐old	DNA, CitH3, F4/80	[63]
Atherosclerotic	Atherosclerotic plaques of autopsied patients who had died of acute myocardial infarction (MI)		CD68, MPO, CitH3	[3]
Pancreatic ductal adenocarcinoma (PDAC)	RAW 264.7 cells, myeloid cell lines THP1	Indirectly coculturing with tumor cells (human PDAC cells) for 72 h	DNA, H3, CitH3, MPO, MMP2, MMP9, MMP12	[14]
Sorafenib	Mouse bone marrow‐derived macrophage (mBMDM), THP‐1‐derived macrophages	Cultured in the conditioned medium (CM) from sorafenib‐treated HCC cells, 12 h	DNA, MPO, CitH3, F4/80	[67]
Glioblastoma	Formalin‐fixed paraffin‐embedded blocks of glioblastoma		CitH3, CD163 and CD204 characterize macrophages	[15]

The release of METs is typically accompanied by the secretion of various proteins and inflammatory mediators. Immunofluorescence staining of tissue sections can detect local increases in cytokines such as TNF‐α and IL‐1β, while western blotting is used to identify METs‐associated proteins, including NE, MPO, histone H3, and CitH3 [71]. These proteins are considered general markers of METs. Various MMPs, including MMP12 and MMP9, are also frequently enriched in METs and are often visualized in combination with histone H3 or CitH3 [14]. However, due to significant structural and compositional overlap among different ETs, particularly with NETs, these markers alone cannot definitively identify METs. Therefore, combining ET markers with cell‐specific surface antigens is essential for accurate attribution. Macrophage markers commonly used for this purpose include F4/80, CD68, and CD163. For instance, in a diabetic mouse model, METs were identified by co‐staining for CD68 and CitH3 [170]. Similarly, in studies of atherosclerotic plaques, triple immunostaining for CD68, MPO, and CitH3 allowed clear discrimination that METs were defined as CD68, MPO, and CitH3‐detecting‐positive structures, whereas NETs were CD68‐negative structures, suggesting that the use of macrophage markers co‐localized with marker proteins is an effective way to differentiate between different ETs [3]. It is important to note that macrophage markers may vary with polarization state. M1 macrophages often express CD80, CD86, CD64, CD16, CD32, and iNOS, while M2 macrophages are typically identified by CD163, CD206, and Arg1 [176]. In human glioblastoma studies, METs‐producing macrophages have been further subclassified using Iba1 together with M2 markers (CD163, CD204) or M1 markers (CD80) [15]. Thus, initial screening with pan‐macrophage markers (e.g., F4/80, CD68) can be followed by polarization‐specific staining for functional characterization. Furthermore, cell viability assays, such as CCK‐8 or LDH release, are valuable for determining whether METs release occurs via lytic or non‐lytic pathways [64]. Finally, live‐cell imaging using dyes like PicoGreen or SYTOX Green enables real‐time quantification of extracellular DNA release. Together, these approaches—combining histology, protein detection, cell marker co‐localization, and viability assessment—provide a multidimensional framework for detecting METs, elucidating their release mechanisms, and distinguishing them from other extracellular traps.

#### 5.2.2 Targeting METs in Cancer Immunotherapy

METs play a critical role in immune defense by capturing and restricting pathogen dissemination in physiological conditions [37, 44, 46]. However, dysregulated METs formation perpetuates inflammation and contributes to the pathogenesis of various diseases [3, 68]. In cancer progression, METs exert pro‐tumor and anti‐tumor effects, and may influence the efficacy of cancer immunotherapy. From an immunoregulatory perspective, excess ETs can be degraded via autoimmune mechanisms to maintain homeostasis. Macrophages clear NETs through coordinated extracellular pre‐processing and subsequent phagocytosis, with lysosomal enzyme systems responsible for intracellular degradation of NETs [89]. Nevertheless, the in vivo self‐clearance mechanisms of METs remain poorly understood. Consequently, therapeutic strategies targeting METs formation and clearance show considerable promise. Modulating the levels and state of METs may mitigate their pro‐tumor effects, thereby offering novel combinatorial approaches for tumor immunotherapy.

Based on the mechanisms of METs formation and structural features, current strategies often employ PAD inhibitors (e.g., Cl‐amidine) to inhibit METs generation or DNase I to degrade existing METs. In murine models of fibrotic injury, the role of METs in organ fibrosis progression has been investigated using PAD4 deficiency or DNase 1 [177]. In cancer immunotherapy, modalities such as CAR‐T therapy and immune checkpoint inhibitors require direct contact with cancer cells to exert their effects, a process potentially impeded by the physical barrier formed by METs. Therefore, disrupting METs barriers with PAD inhibitors or DNase I may enhance the penetration and efficacy of immunotherapeutic agents. However, the use of pan‐PAD inhibitors or DNase I may lead to off‐target effects. Beyond METosis, PAD inhibitors have demonstrated significant effects in a range of diseases including RA and nasopharyngeal carcinoma [178, 179]. Clinical application of pan‐PAD inhibitors to modulate METs levels could interfere with other physiological processes, disrupt immune homeostasis, and even induce complications. A viable strategy involves conjugating therapeutic agents with METs‐specific markers to achieve targeted delivery to pathological sites. Currently, METs can be effectively distinguished from other extracellular traps through the detection of universal DNA scaffolds combined with CitH3 and macrophage markers (e.g., CD163, CD68) [3]. Leveraging these identifiers in drug carrier design would enable spatially controlled release at target lesions, greatly lowering the potential for off‐target activity [180]. Additionally, careful optimization of administration protocols is essential to lessen off‐target impacts caused by nonspecific interactions or the accumulation of drug effects over time [180]. Notably, compared to PAD4 inhibitors, PAD2 inhibitors exhibit higher specificity for METs release with minimal perturbation of other regulatory processes such as NETosis [38, 39]. Additionally, non‐PAD‐mediated pathways of METs release exist, indicating mechanistic limitations of solely targeting PAD [26]. Thus, developing agents that target METs‐specific components may enable more precise combination therapies.

The release potential of METs is associated with macrophage polarization phenotypes, and METs derived from different phenotypes may exhibit compositional differences [38]. M1 macrophages are more prone to releasing METs, while only a limited number of studies have reported METs originating from M2 macrophages [66, 67]. Consequently, modulating macrophage polarization states has emerged as a key strategy for intervening in METosis. For example, liraglutide suppresses METs formation in a model of death‐induced renal dysfunction by promoting M2 macrophage polarization, indicating that inducing M2 polarization effectively reduces METs release [69]. In various disease contexts, promoting M2 polarization not only helps reduce METs release but also supports tissue homeostasis and repair. However, within the tumor microenvironment, inducing M2 polarization presents a therapeutic paradox. M2 macrophages often dominate the late stages of tumor progression, fostering an immunosuppressive microenvironment that promotes metastasis and chemoresistance [181]. This significantly undermines the efficacy of various cancer immunotherapies, such as oncolytic virotherapy and CAR‐T, which are designed to enhance host immune responses [182]. Current clinical strategies often combine agents like sorafenib or lenvatinib with other immunotherapeutics to reprogram the tumor microenvironment and enhance anti‐tumor efficacy [183, 184]. Paradoxically, monotherapeutic strategies, such as simply converting M2 macrophages to an M1 phenotype, risk inducing excessive inflammation and aberrantly elevated METs levels. In HCC, elevated MET levels induce a state of sorafenib resistance, which can ultimately lead to tumor escape [67]. Therefore, clinical applications necessitate multi‐targeted, coordinated modulation. One potential strategy involves the combined use of specific PAD enzyme inhibitors (to suppress METs release) with immune checkpoint inhibitors (e.g., anti‐PD‐1 antibodies), thereby synergistically regulating macrophage polarization and enhancing therapeutic precision.

Cancer patients often exhibit compromised peripheral immune systems, manifesting as impaired hematopoiesis and reduced dendritic cell subsets [185, 186]. Under physiological conditions, METs serve as a natural immune barrier by capturing pathogens and limiting their spread [42]. Moreover, in glioblastoma patient cohorts, the presence of METs is associated with a favorable prognosis, suggesting their potential anti‐tumor role [15]. Degrading METs structures directly could disrupt this barrier function, increasing the risk of releasing trapped bacteria and triggering systemic inflammatory responses in cancer therapy. For instance, cancer patients infected with SARS‐CoV‐2 are more susceptible to severe disease and higher mortality [187]. Hence, when designing immune intervention strategies for cancer patients, careful evaluation of the impact on METs function is essential, balancing potential therapeutic benefits against risks of immune dysregulation. In the context of NET targeting, highly biosafe mPDA‐PEI@GelMA hydrogel microspheres have been developed that capture NETs by strongly binding cfDNA structures without fully degrading the DNA backbone, significantly alleviating wound inflammation and promoting healing in diabetic mouse models [174]. This approach offers a new direction for METs intervention; developing similar safe clearance systems may enable precise modulation of METs levels while preserving immune barrier functions.

Rational combination therapy strategies represent a key direction for enhancing the efficacy of tumor immunotherapy. Currently, cancer immunotherapies are often combined with chemotherapy or radiotherapy to synergistically enhance anti‐tumor immune responses [188]. In a Phase III clinical trial for advanced squamous non‐small cell lung cancer (NSCLC), the combination of ivonescimab with chemotherapy extended median progression‐free survival to 11.1 months, surpassing the current standard therapy by 4.2 months [189]. In this context, combining MET inhibitors with cancer immunotherapy holds promise for breaking through the efficacy plateau. Specifically, using DNase to clear METs barriers may convert “cold” tumors into “hot” tumors, enhancing T‐cell infiltration into tumor tissues and thereby improving the efficacy of immune checkpoint inhibitors [190]. Moreover, METs contribute to pancreatic cancer metastasis; reducing MET levels suppresses tumor seeding and metastatic formation, prolonging patient survival. METs induce T‐cell exhaustion, and clearing METs could restore T‐cell cytotoxicity, ideally complementing the “brake release” mechanism of immune checkpoint inhibitors. To improve drug targeting and therapeutic outcomes, the development of various co‐delivery systems, such as STING agonist delivery systems and radiosensitizer delivery systems, has shown significant progress [191, 192]. Constructing specific co‐delivery platforms could enable precise accumulation of METs‐targeting agents and immunotherapeutics at tumor sites, enhancing efficacy and reducing systemic toxicity. In the SCI‐related inflammation, a biomimetic nanoplatform (DHCNPs) has been developed, featuring a curcumin‐loaded liposome core encapsulated with a fused membrane coating to deliver DNase I for targeted NETs degradation and barrier repair [193]. This demonstrates the considerable potential of constructing targeted delivery platforms for METs intervention. Nanocarriers designed to actively target macrophages (e.g., via CD206) or lesion sites could enable precise delivery of PAD4 inhibitors or polarization modulators, potentially reducing off‐target effects [194]. However, nanocarrier selection involves critical trade‐offs. Inorganic materials (e.g., Se nanoparticles) may exhibit intrinsic anti‐tumor activity but face challenges in biodegradation; organic systems (e.g., liposomes) offer better biocompatibility but often suffer from low drug loading and poor stability. In translation, the choice must be context‐specific. In practical clinical translation, the choice requires careful weighing based on specific application scenarios. Furthermore, the delivery efficiency of nanomedicines and their ability to overcome barriers posed by tumor heterogeneity remain critical unresolved issues [195].

Therefore, research on targeted modulation of METs remains in its nascent stages. Current strategies primarily focus on inhibiting METs formation using PAD enzyme inhibitors (e.g., Cl‐amidine) or promoting their structural degradation via DNase I. Modulating macrophage polarization toward the M2 phenotype is regarded as a potential approach to reduce METs release. However, monotherapy targeting METs shows limited efficacy within the complex tumor immune microenvironment. Combining METs‐directed strategies with cancer immunotherapies (e.g., immune checkpoint inhibitors, CAR‐T) may yield synergistic anti‐tumor efficacy through complementary mechanisms. Notably, direct and specific targeting of MET components remains largely unexplored. Future efforts should focus on elucidating context‐specific MET mechanisms in cancer and developing selective, safe interventions to advance clinical translation.

## 6 Current Challenges and Where the Field is Going

METs consist of a DNA backbone decorated with various antimicrobial proteins. Histone citrullination, a hallmark of many extracellular traps, is generally mediated by PAD. However, the discovery of a non‐PAD‐dependent pathway for METosis, which raises the key question of whether citrullinated histones are present in METs released via the PAD‐independent pathway [26]. A comparative analysis of the structural composition of METs produced through classical PAD‐dependent versus non‐canonical pathways is therefore warranted. Moreover, the propensity for METs release also varies with macrophage polarization state. M1‐polarized macrophages are the primary source of METs, whereas M2 macrophages demonstrate a limited capacity for METosis [66, 67]. It remains unclear whether METs derived from different macrophage subsets exhibit distinct structural or functional properties, which may underlie the context‐dependent, “double‐edged” role of METs in cancer development. Deciphering the specific mechanisms and signals that drive stimulus‐induced METosis is critical to advancing targeted METs drug development for cancer therapy.

Current detection of METs primarily relies on co‐localization of universal ETs markers (e.g., citrullinated histones) with macrophage‐specific markers [15, 170]. A deeper understanding of the structural heterogeneity of METs originating from different pathways would directly enhance the specificity and accuracy of these assays. It is important to note that existing methodologies have significant limitations. Techniques such as immunofluorescence, which assess protein and DNA distribution, often lack objective quantification. Moreover, detection of extracellular DNA may yield false‐positive results due to contamination from other cellular sources. While electron microscopy provides detailed ultrastructural information, it cannot dynamically track METs formation or interaction in real time. Future efforts should therefore prioritize the development of high‐resolution, real‐time imaging technologies to visualize the spatiotemporal dynamics of ETs formation, function, and clearance within native immune environments.

Precisely delineating the pathogenic contributions of ETs in complex diseases, particularly cancer, remains a major challenge. Research on METs in disease contexts is still in its exploratory phase compared to other ETs, such as NETs. METs have been identified in multiple sterile inflammatory diseases, including fibrotic injury, yet significant knowledge gaps remain regarding their mechanistic roles in cancer progression [71]. Current evidence indicates that in solid tumors such as PDAC and CRC, METs facilitate tumor invasion and metastasis by degrading the ECM [12, 14]. However, a retrospective analysis of glioblastoma patients revealed better prognosis in cases with METosis, suggesting potential anti‐tumor activity of METs [15]. These paradoxical findings highlight the complex functions of METs in the TME, though the precise regulatory mechanisms remain elusive. A deeper exploration of METs' potential in promoting and suppressing tumors is critical for developing targeted therapeutic strategies.

Notably, the high heterogeneity of the TME profoundly influences the functional states of TAMs, and METs released further contribute to shaping an immunosuppressive TME. However, the “molecular switches” that regulate METs' function within the dynamically evolving TME remain unclear. While cancer immunotherapy has achieved transformative success in hematologic malignancies (e.g., leukemia, lymphoma), its efficacy against solid tumors remains limited, hampered by profoundly immunosuppressive TMEs, target heterogeneity, and inadequate immune cell infiltration [196]. In PDAC, METosis actively contributes to the establishment of an immunosuppressive TME [14]. Consequently, the development of novel agents specifically targeting METs holds remarkable promise for reprogramming the immunological state of the TME, potentially enhancing T‐cell recruitment and activation. This approach offers a strategic avenue for improving responses to immunotherapy in solid tumors. Therefore, deciphering the bidirectional crosstalk between the TME and METosis is crucial for understanding immune evasion and developing novel therapeutic modalities.

Upon stimulation by certain pathogens, METs can be trained as a memory response, resulting in a stronger release of METs during secondary infections [48, 197]. T cell memory is fundamental for achieving long‐term survival and preventing relapse in cancer patients, with the conversion of stem‐like exhausted T cells into memory subsets being a critical objective of immunotherapy [198]. In contrast, METs exhibit a trained immunity that may have negative effects in cancer. In infection settings, METs' memory could enhance host defense and improve protection [48, 197]. However, in conditions where excessive METs contribute to pathogenesis, such as systemic inflammatory autoimmune diseases or METs‐facilitated tumor metastasis, a secondary stimulus triggering amplified METs release may lead to uncontrolled inflammation, disease exacerbation, and even counteract the efficacy of cancer immunotherapies. Consequently, targeted regulation of the METs memory effect may represent an important future direction in the study of extracellular immune memory.

Currently, substantial gaps remain in the development of targeted therapeutic strategies for modulating METs. From a homeostatic standpoint, investigating alternative ETs‐degrading enzymes beyond DNase I could provide critical insights into how the body efficiently clears METs while avoiding sustained inflammatory responses. Another key direction for future research involves the design of specific pharmacological agents and advanced delivery systems. By harnessing emerging technologies, it may be possible to engineer monoclonal antibodies, peptides, or functionalized nanomaterials that selectively bind and neutralize pathogenic ET components. Moreover, organoid models that more faithfully recapitulate the human tissue microenvironment represent promising platforms for elucidating the mechanisms of METs formation, function, and the tissue‐specific efficacy of targeted interventions.

## 7 Conclusion

**FIGURE 7:**
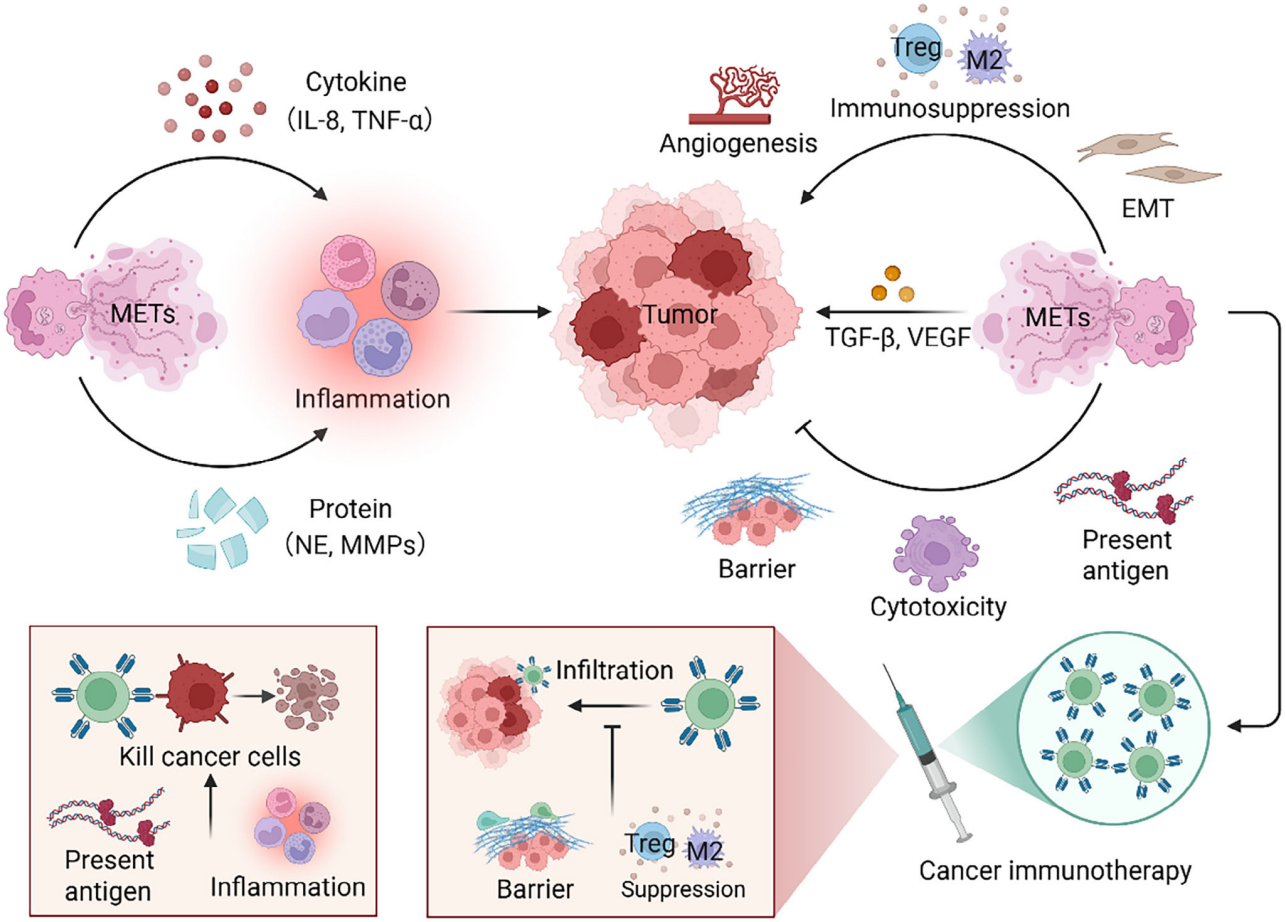
METs in immunity and cancer. By amplifying inflammatory signaling through the release of diverse cytokines and proteins, METs perpetuate chronic inflammation. During subsequent cancer development, METs promote tumor growth by inducing an immunosuppressive microenvironment and stimulating angiogenesis. METs also participate in key immunological processes such as forming physical barriers and modulating antigen presentation, collectively exerting complex influences on cancer progression and immunotherapy efficacy. Created in https://BioRender.com.

METs represent an effector mechanism of macrophage‐mediated immunity, demonstrating a crucial role across various physiological processes (Figure 7). Their release is triggered by diverse stimuli, including chemical agents and pathogens, primarily through inflammatory pathways such as TLR signaling. Upon pathogen invasion, METs entrap, kill, and restrict microbial dissemination, albeit in a non‐specific manner. However, dysregulated METosis contributes to the pathogenesis of multiple diseases. METs release is accompanied by pro‐inflammatory cytokines such as IL‐8 and TNF‐α, along with autoantigen exposure that drives excessive inflammation. In addition, METs‐associated proteins, including MMPs, induce epithelial damage and extracellular matrix degradation. These mechanisms exacerbate the progression of neuroinflammation, organ fibrosis, and autoimmune disorders.

As a leading cause of global mortality, cancer represents a major focus of METs research. Although still in its early stages, evidence points to the potential of METs in tumor progression. METs‐induced chronic inflammation serves as a cancer trigger, while METs‐derived factors such as MMPs and EGF promote metastasis via ECM degradation and angiogenesis—as demonstrated in PDAC and CC. Thus, METs may serve as prognostic biomarkers in certain cancer types. Conversely, in glioblastoma, METs presence correlates with a favorable patient prognosis, suggesting context‐dependent anti‐tumor effects likely influenced by TME heterogeneity and macrophage subsets. Notably, METs exhibit bidirectional immunomodulatory potential in cancer immunotherapy, capable of either synergizing with or antagonizing therapeutic responses. Therefore, elucidating the mechanistic basis of METs in cancer and identifying molecular switches governing their functional polarity holds significant scientific value.

Given the pivotal role of METs in disease pathogenesis, targeting their regulation presents considerable therapeutic promise. Current strategies often employ PAD inhibitors to suppress METs formation or DNase I to facilitate their degradation. Modulating macrophage polarization also serves as an effective approach to regulate METosis, with M1 macrophages exhibiting high METs‐release potential. Nevertheless, the development of selective MET‐targeting agents remains an area of substantial unmet need. Creating therapeutics that mitigate METs‐driven inflammation or synergize with cancer immunotherapy to suppress metastasis carries important clinical implications. Future advances will likely rely on interdisciplinary integration and emerging technologies, particularly nanotechnology, to enable precise METs modulation and achieve therapeutic breakthroughs.

## Author Contributions

J.L. drafted the manuscript. Q.W., A.G., L.P., E.N., and K.K. critically revised the article for important intellectual content and provided financial help. All authors read and approved the final manuscript.

## Funding

This work was supported by Hubei Science and Technology Plan Project (2025EHA016). Also supported by the projects—Excellence PrF UHK 2208/2024–2025, MH CZ—DRO (UHHK, 00179906), CZ.10.03.01/00/22_003/0000048, and Excellence FIM UHK 2203. This paper was created as part of the project No.CZ.02.01.01/00/22_008/000463. Materials and technologies for sustainable development within the Jan Amos Komensky Operational Program financed by the European Union and from the state budget of the Czech Republic.

## Conflicts of Interest

The authors declare that the research was conducted in the absence of any commercial or financial relationships that could be construed as a potential conflict of interest.

## Data Availability

The data that support the findings of this study are available from the corresponding author upon reasonable request.

## References

[1] S. Chen , A. Saeed , Q. Liu , et al., “Macrophages in Immunoregulation and Therapeutics,” Signal Transduction and Targeted Therapy 8, no. 1 (2023): 207, 10.1038/s41392-023-01452-1. 37211559 PMC10200802

[2] V. Brinkmann , U. Reichard , C. Goosmann , et al., “Neutrophil Extracellular Traps Kill Bacteria,” Science 303, no. 5663 (2004): 1532–1535, 10.1126/science.1092385. 15001782

[3] K. R. Pertiwi , O. J. de Boer , C. Mackaaij , et al., “Extracellular Traps Derived from Macrophages, Mast Cells, Eosinophils and Neutrophils are Generated in a Time‐Dependent Manner During Atherothrombosis,” The Journal of Pathology 247, no. 4 (2019): 505–512, 10.1002/path.5212. 30506885 PMC6590313

[4] H. Zhang , J. Liu , Y. Zhou , et al., “Neutrophil Extracellular Traps Mediate m (6) A Modification and Regulates Sepsis‐Associated Acute Lung Injury by Activating Ferroptosis in Alveolar Epithelial Cells,” International Journal of Biological Sciences 18, no. 8 (2022): 3337–3357, 10.7150/ijbs.69141. 35637949 PMC9134924

[5] L. Yang , Q. Liu , X. Zhang , et al., “DNA of Neutrophil Extracellular Traps Promotes Cancer Metastasis via CCDC25,” Nature 583, no. 7814 (2020): 133–138, 10.1038/s41586-020-2394-6. 32528174

[6] L. J. O'Neil , C. B. Oliveira , X. Wang , et al., “Neutrophil Extracellular Trap‐associated Carbamylation and Histones Trigger Osteoclast Formation in Rheumatoid Arthritis,” Annals of the Rheumatic Diseases 82, no. 5 (2023): 630–638, 10.1136/ard-2022-223568. 36737106 PMC11302494

[7] S. Zheng , A. B. Kummarapurugu , G. B. Bulut , A. Syed , L. Kang , and J. A. Voynow , “Neutrophil Elastase Activates the Release of Extracellular Traps from COPD Blood Monocyte‐Derived Macrophages,” Clinical and Translational Science 16, no. 12 (2023): 2765–2778, 10.1111/cts.13671. 37926919 PMC10719474

[8] M. Zhai , S. Gong , P. Luan , et al., “Extracellular Traps from Activated Vascular Smooth Muscle Cells Drive the Progression of Atherosclerosis,” Nature Communications 13, no. 1 (2022): 7500, 10.1038/s41467-022-35330-1. PMC9723654 36473863

[9] L. Maiorino , J. Daßler‐Plenker , L. Sun , et al., “Innate Immunity and Cancer Pathophysiology,” Annual Reviews of Pathology 2022, 17, 425, 10.1146/annurev-pathmechdis-032221-115501. PMC9012188 34788549

[10] X. Li , P. Ramadori , D. Pfister , M. Seehawer , L. Zender , and M. Heikenwalder , “The Immunological and Metabolic Landscape in Primary and Metastatic Liver Cancer,” Nature Reviews Cancer 21, no. 9 (2021): 541–557, 10.1038/s41568-021-00383-9. 34326518

[11] M. Schmitt and F. R. Greten , “The Inflammatory Pathogenesis of Colorectal Cancer,” Nature Reviews Immunology 21, no. 10 (2021): 653–667, 10.1038/s41577-021-00534-x. 33911231

[12] T. Chen , Y. Wang , Z. Nan , et al., “Interaction Between Macrophage Extracellular Traps and Colon Cancer Cells Promotes Colon Cancer Invasion and Correlates with Unfavorable Prognosis,” Frontiers in Immunology 12 (2021): 779325, 10.3389/fimmu.2021.779325. 34925357 PMC8671452

[13] L. Cao , C. Huang , D. Cui Zhou , et al., “Proteogenomic Characterization of Pancreatic Ductal Adenocarcinoma,” Cell 184, no. 19 (2021): 5031–5052.e26, 10.1016/j.cell.2021.08.023. 34534465 PMC8654574

[14] C.‐Y. Liao , G. Li , F.‐P. Kang , et al., “Necroptosis Enhances ‘Don't Eat Me’ signal and Induces Macrophage Extracellular Traps to Promote Pancreatic Cancer Liver Metastasis,” Nature Communications 15, no. 1 (2024): 6043, 10.1038/s41467-024-50450-6. PMC11258255 39025845

[15] A. Michiba , K. Shiogama , T. Tsukamoto , M. Hirayama , S. Yamada , and M. Abe , “Morphologic Analysis of M2 Macrophage in Glioblastoma: Involvement of Macrophage Extracellular Traps (METs),” Acta Histochemica Et Cytochemica 55, no. 4 (2022): 111–118, 10.1267/ahc.22-00018. 36060293 PMC9427541

[16] S. Tan , D. Li , and X. Zhu , “Cancer Immunotherapy: Pros, Cons and beyond,” Biomedicine & Pharmacotherapy 124 (2020): 109821, 10.1016/j.biopha.2020.109821. 31962285

[17] A. Ruiz‐Patiño , O. Arrieta , A. F. Cardona , et al., “Immunotherapy at Any Line of Treatment Improves Survival in Patients with Advanced Metastatic Non‐Small Cell Lung Cancer (NSCLC) Compared with Chemotherapy (Quijote‐CLICaP),” Thoracic Cancer 11, no. 2 (2020): 353–361, 10.1111/1759-7714.13272. 31828967 PMC6996989

[18] L. B. Kennedy and A. K. S. Salama , “A Review of Cancer Immunotherapy Toxicity,” CA: A Cancer Journal for Clinicians 70, no. 2 (2020): 86, 10.3322/caac.21596. 31944278

[19] A. Loureiro , C. Pais , and P. Sampaio , “Relevance of Macrophage Extracellular Traps in C. albicans Killing,” Frontiers in Immunology 10 (2019): 2767, 10.3389/fimmu.2019.02767. 31866996 PMC6904331

[20] M. Jensen , N. W. Thorsen , L. A. E. Hallberg , P. Hägglund , and C. L. Hawkins , “New Insight into the Composition of Extracellular Traps Released by Macrophages Exposed to Different Types of Inducers,” Free Radical Biology and Medicine 202 (2023): 97–109, 10.1016/j.freeradbiomed.2023.03.025. 36990299

[21] D. Drab , M. Santocki , M. Opydo , and E. Kolaczkowska , “Impact of Endogenous and Exogenous Nitrogen Species on Macrophage Extracellular trap (MET) Formation by Bone Marrow–Derived Macrophages,” Cell and Tissue Research 394, no. 2 (2023): 361–377, 10.1007/s00441-023-03832-z. 37789240 PMC10638184

[22] R. S. Doster , L. M. Rogers , J. A. Gaddy , and D. M. Aronoff , “Macrophage Extracellular Traps: A Scoping Review,” Journal of Innate Immunity 10, no. 1 (2018): 3–13, 10.1159/000480373. 28988241 PMC6757166

[23] C. T. Robb , E. A. Dyrynda , R. D. Gray , A. G. Rossi , and V. J. Smith , “Invertebrate Extracellular Phagocyte Traps Show That Chromatin Is an Ancient Defence Weapon,” Nature Communications 5 (2014): 4627, 10.1038/ncomms5627. PMC4143918 25115909

[24] S. Kalsum , C. Braian , V. A. C. M. Koeken , et al., “The Cording Phenotype of Mycobacterium Tuberculosis Induces the Formation of Extracellular Traps in Human Macrophages,” Frontiers in Cellular and Infection Microbiology 7 (2017): 278, 10.3389/fcimb.2017.00278. 28695112 PMC5483443

[25] B. S. Rayner , B. E. Brown , Y. Zhang , V. C. Cogger , and C. L. Hawkins , “The Myeloperoxidase‐Derived Oxidant Hypochlorous Acid Induces Macrophage Extracellular Trap Release and Promotes inflammation,” Free Radical Biology and Medicine 96 (2016): S37–S38, 10.1016/j.freeradbiomed.2016.04.075.

[26] B. S. Rayner , Y. Zhang , B. E. Brown , L. Reyes , V. C. Cogger , and C. L. Hawkins , “Role of Hypochlorous Acid (HOCl) and Other Inflammatory Mediators in the Induction of Macrophage Extracellular Trap Formation,” Free Radical Biology and Medicine 129 (2018): 25–34, 10.1016/j.freeradbiomed.2018.09.001. 30189264

[27] K. W. Wong and W. R. Jacobs , “Mycobacterium Tuberculosis Exploits human Interferon γ to Stimulate Macrophage Extracellular Trap Formation and Necrosis,” The Journal of Infectious Diseases 208, no. 1 (2013): 109–119, 10.1093/infdis/jit097. 23475311 PMC3666134

[28] X. Zhao , X. Tang , N. Guo , et al., “Biochanin a Enhances the Defense against Salmonella Enterica Infection through AMPK/ULK1/mTOR‐Mediated Autophagy and Extracellular Traps and Reversing SPI‐1‐Dependent Macrophage (MΦ) M2 Polarization,” Frontiers in Cellular and Infection Microbiology 8 (2018): 318, 10.3389/fcimb.2018.00318. 30271755 PMC6142880

[29] M. Oda , M. Kurosawa , H. Yamamoto , et al., “Sulfated Vizantin Induces Formation of Macrophage Extracellular Traps,” Microbiology and Immunology 62, no. 5 (2018): 310–316, 10.1111/1348-0421.12589. 29577412

[30] Y. Liu , J. Liang , J.‐W. Li , et al., “Phagocyte Extracellular Traps Formation Contributes to Host Defense against Clostridium Perfringens Infection,” Cytokine 169 (2023): 156276, 10.1016/j.cyto.2023.156276. 37339556

[31] Y. Sun , B. Li , B. Song , et al., “CREB1/CRTC2 Regulated Tubular Epithelial‐derived Exosomal miR‐93‐3p Promotes Kidney Injury Induced by Calcium Oxalate via Activating M1 Polarization and Macrophage Extracellular Trap Formation,” Journal of Nanobiotechnology 23, no. 1 (2025): 204, 10.1186/s12951-025-03246-9. 40069788 PMC11900527

[32] Q. Feng , J. Yuan , Y. Hu , et al., “Macrophage Extracellular Traps as Key Mediators of Scleral Remodeling in Myopia Induced by Hypoxia and Activated Platelets,” Cell Reports 44, no. 6 (2025): 115771, 10.1016/j.celrep.2025.115771. 40440172

[33] A. B. Kummarapurugu , S. Zheng , J. Ma , S. Ghosh , A. Hawkridge , and J. A. Voynow , “Neutrophil Elastase Triggers the Release of Macrophage Extracellular Traps: Relevance to Cystic Fibrosis,” American Journal of Respiratory Cell and Molecular Biology 66, no. 1 (2022): 76–85, 10.1165/rcmb.2020-0410OC. 34597246 PMC8803356

[34] O. A. Chow , M. von Köckritz‐Blickwede , A. T. Bright , et al., “Statins Enhance Formation of Phagocyte Extracellular Traps,” Cell Host & Microbe 8, no. 5 (2010): 445–454, 10.1016/j.chom.2010.10.005. 21075355 PMC3008410

[35] S. Tonello , F. Carniato , M. Rizzi , et al., “Charged Polyhedral Oligomeric Silsesquioxanes Trigger in Vitro METosis via both Oxidative Stress and Autophagy,” Life Sciences 190 (2017): 58–67, 10.1016/j.lfs.2017.09.040. 28966135

[36] D. Luo , J. Zhang , H. Yin , S. Li , S. Xu , and S. Li , “Cannabidiol Alleviates Perfluorooctane Sulfonate‐induced Macrophage Extracellular Trap Mediate Inflammation and Fibrosis in Mice Liver,” Ecotoxicology and Environmental Safety 263 (2023): 115374, 10.1016/j.ecoenv.2023.115374. 37591127

[37] K. Yin , D. Wang , Y. Zhang , et al., “Polystyrene Microplastics Promote Liver Inflammation by Inducing the Formation of Macrophages Extracellular Traps,” Journal of Hazardous Materials 452 (2023): 131236, 10.1016/j.jhazmat.2023.131236. 36958159

[38] N. Mishra , M. Mohs , N. Wittmann , S. Gross , P. R. Thompson , and L. Bossaller , “PLC and PAD2 Regulate Extracellular Calcium‐Triggered Release of Macrophage Extracellular DNA Traps,” European Journal of Immunology 55, no. 4 (2025): 202350942, 10.1002/eji.202350942. PMC11962252 40170382

[39] S. J. Bashar , C. L. Holmes , and M. A. Shelef , “Macrophage Extracellular Traps Require Peptidylarginine Deiminase 2 and 4 and Are a Source of Citrullinated Antigens Bound by Rheumatoid Arthritis Autoantibodies,” Frontiers in Immunology 15 (2024): 1167362, 10.3389/fimmu.2024.1167362. 38476240 PMC10927735

[40] L. Pijanowski , M. Scheer , B. M. L. Verburg‐van Kemenade , and M. Chadzinska , “Production of Inflammatory Mediators and Extracellular Traps by Carp Macrophages and Neutrophils in Response to Lipopolysaccharide and/or Interferon‐γ2,” Fish & Shellfish Immunology 42, no. 2 (2015): 473–482, 10.1016/j.fsi.2014.11.019. 25453727

[41] T. Bamra , T. Shafi , S. Das , M. Kumar , and P. Das , “Leishmania Donovani Mevalonate Kinase Regulates Host Actin for Inducing Phagocytosis,” Biochimie 220 (2024): 31–38, 10.1016/j.biochi.2023.12.003. 38123120

[42] A. A. Baz , S. Chen , H. Hao , et al., “Macrophage Extracellular Traps are Induced by Mycoplasma Bovis in Bovine Macrophages through NADPH Oxidase/ ROS ‐Dependent Manner and Their Antibacterial Efficacy,” The FASEB Journal 38, no. 23 (2024): 70238, 10.1096/fj.202402304R. PMC11627205 39652064

[43] L. Li , X. Li , G. Li , et al., “Mouse Macrophages Capture and Kill Giardia lamblia by Means of Releasing Extracellular Trap,” Developmental & Comparative Immunology 88 (2018): 206–212, 10.1016/j.dci.2018.07.024. 30048699

[44] Y. Liao , Z. Zhu , Y. Liu , et al., “Schistosome Egg‐Derived Extracellular Vesicles Deliver Sja‐miR‐71a Inhibits Host Macrophage and Neutrophil Extracellular Traps via Targeting Sema4D,” Cell Communication and Signaling 21, no. 1 (2023): 366, 10.1186/s12964-023-01395-8. 38129877 PMC10734185

[45] Z. Wei , Y. Wang , X. Zhang , et al., “Bovine Macrophage‐Derived Extracellular Traps Act as Early Effectors against the Abortive Parasite Neospora Caninum,” Veterinary Parasitology 258 (2018): 1–7, 10.1016/j.vetpar.2018.06.002. 30105969

[46] P. Liu , X. Wu , C. Liao , et al., “Escherichia Coli and Candida Albicans Induced Macrophage Extracellular Trap‐like Structures with Limited Microbicidal Activity,” PLoS ONE 9, no. 2 (2014): 90042, 10.1371/journal.pone.0090042. PMC3934966 24587206

[47] A. Mónaco , N. Canales‐Huerta , J. Jara‐Wilde , et al., “Salmonella Typhimurium Triggers Extracellular Traps Release in Murine Macrophages,” Frontiers in Cellular and Infection Microbiology 11 (2021): 639768, 10.3389/fcimb.2021.639768. 33981627 PMC8107695

[48] Y.‐J. Liu , J.‐L. Chen , Z.‐B. Fu , Y. Wang , X.‐Z. Cao , and Y. Sun , “Enhanced Responsive Formation of Extracellular Traps in Macrophages Previously Exposed to Porphyromonas gingivalis,” Inflammation 45, no. 3 (2022): 1174–1185, 10.1007/s10753-021-01611-y. 35059922

[49] N. A. Aulik , K. M. Hellenbrand , and C. J. Czuprynski , “Mannheimia Haemolytica and Its Leukotoxin Cause Macrophage Extracellular Trap Formation by Bovine Macrophages,” Infection and Immunity 80, no. 5 (2012): 1923–1933, 10.1128/iai.06120-11. 22354029 PMC3347434

[50] R. S. Doster , J. A. Sutton , L. M. Rogers , D. M. Aronoff , and J. A. Gaddy , “Streptococcus Agalactiae Induces Placental Macrophages To Release Extracellular Traps Loaded with Tissue Remodeling Enzymes via an Oxidative Burst‐Dependent Mechanism,” MBio 9, no. 6 (2018), 10.1128/mBio.02084-18. PMC6247082 30459195

[51] M. Qian , K. Xu , M. Zhang , et al., “5′‐Nucleotidase is Dispensable for the Growth of Salmonella Typhimurium but inhibits the Bactericidal Activity of Macrophage Extracellular Traps,” Archives of Microbiology 205, no. 1 (2022): 20, 10.1007/s00203-022-03353-3. 36482126

[52] C. M. Romo‐Barrera , L. E. Castrillón‐Rivera , A. Palma‐Ramos , et al., “Bacillus Licheniformis and Bacillus Subtilis, Probiotics That Induce the Formation of Macrophage Extracellular Traps,” Microorganisms 9, no. 10 (2021), 10.3390/microorganisms9102027. PMC8540962 34683348

[53] L. Li , Q. Jiao , Q. Yang , et al., “A Bladder‐Blood Immune Barrier Constituted by Suburothelial Perivascular Macrophages Restrains Uropathogen Dissemination,” Immunity 58, no. 3 (2025): 568–584.e6, 10.1016/j.immuni.2025.02.002. 40015270

[54] Z.‐Z. Liu , W. Chen , C.‐K. Zhou , K. Ma , Y. Gao , and Y.‐J. Yang , “Stimulator of Interferon Genes (STING) Promotes Staphylococcus aureus‐Induced Extracellular Traps Formation via the ROS‐ERK Signaling Pathway,” Frontiers in Cell and Developmental Biology 10 (2022): 836880, 10.3389/fcell.2022.836880. 35399524 PMC8984202

[55] K. Okubo , M. Kurosawa , M. Kamiya , et al., “Macrophage Extracellular Trap Formation Promoted by Platelet Activation Is a Key Mediator of Rhabdomyolysis‐induced Acute Kidney Injury,” Nature Medicine 24, no. 2 (2018): 232–238, 10.1038/nm.4462. 29309057

[56] S. Wu , J. Yang , G. Sun , et al., “Macrophage Extracellular Traps Aggravate Iron Overload‐Related Liver Ischaemia/Reperfusion Injury,” British Journal of Pharmacology 178, no. 18 (2021): 3783–3796, 10.1111/bph.15518. 33959955

[57] S. Je , H. Quan , Y. Yoon , Y. Na , B.‐J. Kim , and S. H. Seok , “Mycobacterium Massiliense Induces Macrophage Extracellular Traps with Facilitating Bacterial Growth,” PLoS ONE 11, no. 5 (2016): 0155685, 10.1371/journal.pone.0155685. PMC4871462 27191593

[58] C. T. Madreiter‐Sokolowski , C. Thomas , and M. Ristow , “Interrelation Between ROS and Ca(2+) in Aging and Age‐Related Diseases,” Redox Biology 36 (2020): 101678, 10.1016/j.redox.2020.101678. 32810740 PMC7451758

[59] D. Azzouz , M. A. Khan , and N. Palaniyar , “ROS Induces NETosis by Oxidizing DNA and Initiating DNA Repair,” Cell Death Discovery 7, no. 1 (2021): 113, 10.1038/s41420-021-00491-3. 34001856 PMC8128883

[60] K. D. Metzler , C. Goosmann , A. Lubojemska , A. Zychlinsky , and V. Papayannopoulos , “A Myeloperoxidase‐containing Complex Regulates Neutrophil Elastase Release and Actin Dynamics during NETosis,” Cell Reports 8, no. 3 (2014): 883–896, 10.1016/j.celrep.2014.06.044. 25066128 PMC4471680

[61] Y. An , X. Shi , X. Tang , et al., “Aflatoxin B1 Induces Reactive Oxygen Species‐Mediated Autophagy and Extracellular Trap Formation in Macrophages,” Frontiers in Cellular and Infection Microbiology 7 (2017): 53, 10.3389/fcimb.2017.00053. 28280716 PMC5322174

[62] X. Liu , T. Arfman , K. Wichapong , C. P. M. Reutelingsperger , J. Voorberg , and G. A. F. Nicolaes , “PAD4 Takes Charge During Neutrophil Activation: Impact of PAD4 Mediated NET Formation on Immune‐Mediated Disease,” Journal of Thrombosis and Haemostasis 19, no. 7 (2021): 1607–1617, 10.1111/jth.15313. 33773016 PMC8360066

[63] Y. Shen , R. Shi , S. Lu , et al., “Role of Peptidyl Arginine Deiminase 4‐Dependent Macrophage Extracellular Trap Formation in Type 1 Diabetes Pathogenesis,” Diabetes 2024, 73 (11), 1862, 10.2337/db23-1000. 39137121

[64] A. S. Carvalho , G. C. Pereira‐Silva , J. M. P. Andrade , et al., “DNA Extracellular Traps Released by Mayaro Virus‐Infected Macrophages Act as a Platform for Virus Dissemination,” Journal of Medical Virology 97, no. 3 (2025): 70262, 10.1002/jmv.70262. 40007117

[65] G. Morris , M. Gevezova , V. Sarafian , and M. Maes , “Redox Regulation of the Immune Response,” Cellular & Molecular Immunology 19, no. 10 (2022): 1079–1101, 10.1038/s41423-022-00902-0. 36056148 PMC9508259

[66] D. Nakazawa , H. Shida , Y. Kusunoki , et al., “The Responses of Macrophages in Interaction with Neutrophils That Undergo NETosis,” Journal of Autoimmunity 67 (2016): 19–28, 10.1016/j.jaut.2015.08.018. 26347075

[67] X. Huang , N. Yi , P. Zhu , J. Gao , and J. Lv , “Sorafenib‐induced Macrophage Extracellular Traps via ARHGDIG/IL4/PADI4 Axis Confer Drug Resistance through Inhibiting Ferroptosis in Hepatocellular Carcinoma,” Biology Direct 19, no. 1 (2024): 110, 10.1186/s13062-024-00560-4. 39529192 PMC11555812

[68] C. Zhang , D. Guo , H. Qiao , et al., “Macrophage Extracellular Traps Exacerbate Secondary Spinal Cord Injury by Modulating Macrophage/Microglia Polarization via LL37/P2X7R/NF‐ κ B Signaling Pathway,” Oxidative Medicine and Cellular Longevity 2022 (2022): 9197940, 10.1155/2022/9197940. 36466087 PMC9713475

[69] Z. Sun , F. Zhang , Z. Gao , et al., “Liraglutide Alleviates Ferroptosis in Renal Ischemia Reperfusion Injury via Inhibiting Macrophage Extracellular Trap Formation,” International Immunopharmacology 142 (2024): 113258, 10.1016/j.intimp.2024.113258. 39340991

[70] C. Lu , Z. Wu , H. Gao , et al., “Sperm Induce Macrophage Extracellular Trap Formation via Phagocytosis‐dependent Mechanism,” Biology of Reproduction 109, no. 3 (2023): 319–329, 10.1093/biolre/ioad068. 37402702

[71] S. Wang , L. Chen , X. Shi , Y. Wang , and S. Xu , “Polystyrene Microplastics‐induced Macrophage Extracellular Traps Contributes to Liver Fibrotic Injury by Activating ROS/TGF‐β/Smad2/3 Signaling Axis,” Environmental Pollution 324 (2023): 121388, 10.1016/j.envpol.2023.121388. 36871749

[72] S. Gao , K. Zheng , J. Lou , et al., “Macrophage Extracellular Traps Suppress Particulate Matter–Induced Airway Inflammation,” The American Journal of Pathology 194, no. 9 (2024): 1622–1635, 10.1016/j.ajpath.2024.05.008. 38897538

[73] N. Song , W. Wang , Y. Wang , Y. Guan , S. Xu , and M.‐Y. Guo , “Hydrogen Sulfide of Air Induces Macrophage Extracellular Traps to Aggravate Inflammatory Injury via the Regulation of miR‐15b‐5p on MAPK and Insulin Signals in Trachea of Chickens,” Science of The Total Environment 771 (2021): 145407, 10.1016/j.scitotenv.2021.145407. 33548704

[74] N. Palaniyar , P. Liu , X. Wu , et al., “Escherichia Coli and Candida albicans Induced Macrophage Extracellular Trap‐Like Structures with Limited Microbicidal Activity,” PLoS ONE 9, no. 2 (2014), 10.1371/journal.pone.0090042. PMC3934966 24587206

[75] W. Weng , Y. Liu , Z. Hu , et al., “Macrophage Extracellular Traps Promote Tumor‐like Biologic Behaviors of Fibroblast‐like Synoviocytes through cGAS‐mediated PI3K/Akt Signaling Pathway in Patients with Rheumatoid Arthritis,” Journal of Leukocyte Biology 115, no. 1 (2024): 116–129, 10.1093/jleuko/qiad102. 37648663

[76] D. E. Da Silva , C. M. Richards , S. A. McRae , et al., “Extracellular Mixed Histones Are Neurotoxic and Modulate Select Neuroimmune Responses of Glial Cells,” PLoS ONE 19, no. 4 (2024): 0298748, 10.1371/journal.pone.0298748. PMC11023449 38630734

[77] D. A. Lomas , “Does Protease‐Antiprotease Imbalance Explain Chronic Obstructive Pulmonary Disease?,” Ann Am Thorac Soc 13 (2016): S130, 10.1513/AnnalsATS.201504-196KV. 27115947

[78] A. S. Bleakley , S. Kho , M. J. Binks , et al., “Extracellular Traps are Evident in Romanowsky‐Stained Smears of Bronchoalveolar Lavage from Children with Non‐Cystic Fibrosis Bronchiectasis,” Respirology 28, no. 12 (2023): 1126–1135, 10.1111/resp.14587. 37648649 PMC10947271

[79] P. T. King , R. Sharma , K. M. O'Sullivan , et al., “Deoxyribonuclease 1 Reduces Pathogenic Effects of Cigarette Smoke Exposure in the Lung,” Scientific Reports 7 (2017): 12128, 10.1038/s41598-017-12474-5. 28935869 PMC5608940

[80] J.‐H. Kim , J.‐W. Kim , C.‐Y. Kim , J.‐S. Jeong , J.‐W. Ko , and T.‐W. Kim , “Green tea extract ameliorates macrophage‐driven emphysematous lesions in chronic obstructive pulmonary disease induced by cigarette smoke condensate,” Phytotherapy Research 37, no. 4 (2023): 1366–1376, 10.1002/ptr.7745. 36729048

[81] F. M. Conedera , D. Kokona , M. S. Zinkernagel , et al., “Macrophages Coordinate Immune Response to Laser‐induced Injury via Extracellular Traps,” Journal of Neuroinflammation 21, no. 1 (2024): 68, 10.1186/s12974-024-03064-0. 38500151 PMC10949579

[82] S.‐A. W. Brown , M. Dobelle , M. Padilla , et al., “Idiopathic Pulmonary Fibrosis and Lung Cancer. A Systematic Review and Meta‐analysis,” Annals of the American Thoracic Society 16 (2019): 1041–1051, 10.1513/AnnalsATS.201807-481OC. 30892054

[83] K. B. Kennel , M. Bozlar , A. F. De Valk , et al., “Cancer‐Associated Fibroblasts in Inflammation and Antitumor Immunity,” Clin Cancer Res 2023, 29 (6), 1009, 10.1158/1078-0432.Ccr-22-1031. 36399325 PMC10011884

[84] A. Bagaev , N. Kotlov , K. Nomie , et al., “Conserved Pan‐cancer Microenvironment Subtypes Predict Response to Immunotherapy,” Cancer Cell 39, no. 6 (2021): 845–865.e7, 10.1016/j.ccell.2021.04.014. 34019806

[85] J. Guinney , R. Dienstmann , X. Wang , et al., “The Consensus Molecular Subtypes of Colorectal Cancer,” Nature Medicine 21, no. 11 (2015): 1350–1356, 10.1038/nm.3967. PMC4636487 26457759

[86] M. E. M. El Shikh , R. El Sayed , A. Nerviani , et al., “Extracellular Traps and PAD4 Released by Macrophages Induce Citrullination and Auto‐antibody Production in Autoimmune Arthritis,” Journal of Autoimmunity 105 (2019): 102297, 10.1016/j.jaut.2019.06.008. 31277965

[87] J. Tang , J. Xia , H. Gao , et al., “IL33‐induced Neutrophil Extracellular Traps (NETs) Mediate a Positive Feedback Loop for Synovial Inflammation and NET Amplification in Rheumatoid Arthritis,” Experimental & Molecular Medicine 56, no. 12 (2024): 2602–2616, 10.1038/s12276-024-01351-7. 39617790 PMC11671579

[88] T. O. Yang , S. Floud , and G. K. Reeves , “Rheumatoid Arthritis and Cancer Risk in the Million Women Study,” International Journal of Epidemiology 53, no. 2 (2024), 10.1093/ije/dyae006. PMC11534085 39495903

[89] P. Haider , J. B. Kral‐Pointner , J. Mayer , et al., “Neutrophil Extracellular Trap Degradation by Differently Polarized Macrophage Subsets,” Arteriosclerosis, Thrombosis, and Vascular Biology 40, no. 9 (2020): 2265–2278, 10.1161/atvbaha.120.314883. 32673525 PMC7447175

[90] X. Xu , Y. Yan , M. Zheng , et al., “Emodin Alleviates Sepsis‐Induced Multiorgan Damage by Inhibiting NETosis through Targeting Neutrophils BCL‐10,” Advanced Science 12, no. 41 (2025): 17129, 10.1002/advs.202417129. PMC12591137 40779122

[91] Y. Xiao and D. Yu , “Tumor Microenvironment as a Therapeutic Target in Cancer,” Pharmacology & Therapeutics 221 (2021): 107753, 10.1016/j.pharmthera.2020.107753. 33259885 PMC8084948

[92] D. G. DeNardo and B. Ruffell , “Macrophages as Regulators of Tumour Immunity and Immunotherapy,” Nature Reviews Immunology 19, no. 6 (2019): 369–382, 10.1038/s41577-019-0127-6. PMC7339861 30718830

[93] I. Vitale , G. Manic , L. M. Coussens , G. Kroemer , and L. Galluzzi , “Macrophages and Metabolism in the Tumor Microenvironment,” Cell Metabolism 30, no. 1 (2019): 36–50, 10.1016/j.cmet.2019.06.001. 31269428

[94] X. Cheng , H. Zhang , A. Hamad , H. Huang , and A. Tsung , “Surgery‐Mediated Tumor‐Promoting Effects on the Immune Microenvironment,” Seminars in Cancer Biology 86 (2022): 408–419, 10.1016/j.semcancer.2022.01.006. 35066156 PMC11770836

[95] T. Luckett , M. Abudula , L. Ireland , et al., “Mesothelin Secretion by Pancreatic Cancer Cells Co‐opts Macrophages and Promotes Metastasis,” Cancer Research 84, no. 4 (2024): 527–544, 10.1158/0008-5472.Can-23-1542. 38356443

[96] X. Zhan , R. Wu , X.‐H. Kong , et al., “Elevated Neutrophil Extracellular Traps by HBV‐Mediated S100A9‐TLR4/RAGE‐ROS Cascade Facilitate the Growth and Metastasis of Hepatocellular Carcinoma,” Cancer Communications 43, no. 2 (2023): 225–245, 10.1002/cac2.12388. 36346061 PMC9926958

[97] I. Melero , M. Villalba‐Esparza , B. Recalde‐Zamacona , et al., “Neutrophil Extracellular Traps, Local IL‐8 Expression, and Cytotoxic T‐Lymphocyte Response in the Lungs of Patients with Fatal COVID‐19,” Chest 162, no. 5 (2022): 1006–1016, 10.1016/j.chest.2022.06.007. 35714708 PMC9197577

[98] Z. Liu , Y. Dou , C. Lu , R. Han , and Y. He , “Neutrophil Extracellular Traps in Tumor Metabolism and Microenvironment,” Biomarker Research 13, no. 1 (2025): 12, 10.1186/s40364-025-00731-z. 39849606 PMC11756210

[99] T. Taifour , S. S. Attalla , D. Zuo , et al., “The Tumor‐derived Cytokine Chi3l1 Induces Neutrophil Extracellular Traps That Promote T Cell Exclusion in Triple‐negative Breast Cancer,” Immunity 56, no. 12 (2023): 2755–2772.e8, 10.1016/j.immuni.2023.11.002. 38039967

[100] J. Li , Y. Xia , B. Sun , et al., “Neutrophil Extracellular Traps Induced by the Hypoxic Microenvironment in Gastric Cancer Augment Tumour Growth,” Cell Communication and Signaling 21, no. 1 (2023): 86, 10.1186/s12964-023-01112-5. 37127629 PMC10152773

[101] Y. Xiao , M. Cong , J. Li , et al., “Cathepsin C Promotes Breast Cancer Lung Metastasis by Modulating Neutrophil Infiltration and Neutrophil Extracellular Trap Formation,” Cancer Cell 39, no. 3 (2021): 423–437, 10.1016/j.ccell.2020.12.012. 33450198

[102] C. Zha , X. Meng , L. Li , et al., “Neutrophil Extracellular Traps Mediate the Crosstalk Between Glioma Progression and the Tumor Microenvironment *via* the HMGB1/RAGE/IL‐8 axis,” Cancer Biology and Medicine 17, no. 1 (2020): 154–168, 10.20892/j.issn.2095-3941.2019.0353. 32296583 PMC7142852

[103] J. Wang , C. Yu , H. Lyu , T. Liu , and R. Xie , “Macrophage Extracellular Traps Promote Peritoneal Fibrosis through the ROS/TGF‐β/Smad Pathway under High‐glucose Dialysis Conditions,” International Immunopharmacology 167 (2025): 115748, 10.1016/j.intimp.2025.115748. 41151485

[104] H. Wang , T. Tian , and J. Zhang , “Tumor‐Associated Macrophages (TAMs) in Colorectal Cancer (CRC): From Mechanism to Therapy and Prognosis,” International Journal of Molecular Sciences 22, no. 16 (2021): 8470, 10.3390/ijms22168470. 34445193 PMC8395168

[105] C. Alfaro , A. Teijeira , C. Oñate , et al., “Tumor‐Produced Interleukin‐8 Attracts Human Myeloid‐Derived Suppressor Cells and Elicits Extrusion of Neutrophil Extracellular Traps (NETs),” Clinical Cancer Research 22, no. 15 (2016): 3924–3936, 10.1158/1078-0432.Ccr-15-2463. 26957562

[106] W. Tang , Z. Chen , W. Zhang , et al., “The Mechanisms of Sorafenib Resistance in Hepatocellular Carcinoma: Theoretical Basis and Therapeutic Aspects,” Signal Transduction and Targeted Therapy 5, no. 1 (2020): 87, 10.1038/s41392-020-0187-x. 32532960 PMC7292831

[107] K. Wu , M. Yan , T. Liu , et al., “Creatine Kinase B Suppresses Ferroptosis by Phosphorylating GPX4 through a Moonlighting Function,” Nature Cell Biology 25, no. 5 (2023): 714–725, 10.1038/s41556-023-01133-9. 37156912

[108] J. P. Friedmann Angeli , D. V. Krysko , and M. Conrad , “Ferroptosis at the Crossroads of Cancer‐Acquired Drug Resistance and Immune Evasion,” Nature Reviews Cancer 19, no. 7 (2019): 405–414, 10.1038/s41568-019-0149-1. 31101865

[109] C. Conche , F. Finkelmeier , M. Pesic , et al., “Combining Ferroptosis Induction with MDSC Blockade Renders Primary Tumours and Metastases in Liver Sensitive to Immune Checkpoint Blockade,” Gut 72, no. 9 (2023): 1774–1782, 10.1136/gutjnl-2022-327909. 36707233 PMC10423492

[110] H. Li , Y. Sun , Y. Yao , et al., “USP8‐Governed GPX4 Homeostasis Orchestrates Ferroptosis and Cancer Immunotherapy,” Proceedings of the National Academy of Sciences U S A 121, no. 16 (2024): 2315541121, 10.1073/pnas.2315541121. PMC11032464 38598341

[111] M. Song , C. Zhang , S. Cheng , et al., “DNA of Neutrophil Extracellular Traps Binds TMCO6 to Impair CD8^+^ T‐cell Immunity in Hepatocellular Carcinoma,” Cancer Research 84, no. 10 (2024): 1613–1629, 10.1158/0008-5472.Can-23-2986. 38381538

[112] W. Huang , W.‐T. Wang , K. Fang , et al., “MIR‐708 Promotes Phagocytosis to Eradicate T‐ALL Cells by Targeting CD47,” Molecular Cancer 17, no. 1 (2018): 12, 10.1186/s12943-018-0768-2. 29368647 PMC5782377

[113] Q. Xi , J. Zhang , G. Yang , et al., “Restoration of miR‐340 Controls Pancreatic Cancer Cell CD47 Expression to Promote Macrophage Phagocytosis and Enhance Antitumor Immunity,” Journal for ImmunoTherapy of Cancer 8, no. 1 (2020): 000253, 10.1136/jitc-2019-000253. PMC7279671 32503944

[114] S. M. P. Vadevoo , Y. Kang , G. R. Gunassekaran , et al., “IL4 Receptor Targeting Enables Nab‐Paclitaxel to Enhance Reprogramming of M2‐type Macrophages into M1‐like Phenotype via ROS‐HMGB1‐TLR4 Axis and Inhibition of Tumor Growth and Metastasis,” Theranostics 14, no. 6 (2024): 2605–2621, 10.7150/thno.92672. 38646639 PMC11024855

[115] M. Chen , Y. Miao , K. Qian , et al., “Detachable Liposomes Combined Immunochemotherapy for Enhanced Triple‐Negative Breast Cancer Treatment through Reprogramming of Tumor‐Associated Macrophages,” Nano Letters 21, no. 14 (2021): 6031–6041, 10.1021/acs.nanolett.1c01210. 34240603

[116] Y. Li , S. Wu , Y. Zhao , et al., “Neutrophil Extracellular Traps Induced by Chemotherapy Inhibit Tumor Growth in Murine Models of Colorectal Cancer,” Journal of Clinical Investigation 134, no. 5 (2024), 10.1172/jci175031. PMC10904055 38194275

[117] F. Schedel , S. Mayer‐Hain , K. I. Pappelbaum , et al., “Evidence and Impact of Neutrophil Extracellular Traps in Malignant Melanoma,” Pigment Cell & Melanoma Research 33, no. 1 (2020): 63–73, 10.1111/pcmr.12818. 31402559

[118] V. Papayannopoulos , “Neutrophil Extracellular Traps in Immunity and Disease,” Nature Reviews Immunology 18, no. 2 (2018): 134–147, 10.1038/nri.2017.105. 28990587

[119] K. H. Rasmussen and C. L. Hawkins , “Role of Macrophage Extracellular Traps in Innate Immunity and Inflammatory Disease,” Biochemical Society Transactions 50, no. 1 (2022): 21–32, 10.1042/bst20210962. 35191493

[120] Y. Zhang , A. Nicolau , C. F. Lima , and L. R. Rodrigues , “Bovine Lactoferrin Induces Cell Cycle Arrest and Inhibits mTOR Signaling in Breast Cancer Cells,” Nutrition and Cancer 66, no. 8 (2014): 1371–1385, 10.1080/01635581.2014.956260. 25356800

[121] Q. Rascón‐Cruz , E. A. Espinoza‐Sánchez , T. S. Siqueiros‐Cendón , et al., “Lactoferrin: A Glycoprotein Involved in Immunomodulation, Anticancer, and Antimicrobial Processes,” Molecules (Basel, Switzerland) 26 (2021), 10.3390/molecules26010205. PMC7795860 33401580

[122] H. Dong , Y. Yang , C. Gao , et al., “Lactoferrin‐containing Immunocomplex Mediates Antitumor Effects by Resetting Tumor‐associated Macrophages to M1 Phenotype,” Journal for ImmunoTherapy of Cancer 8, no. 1 (2020): 000339, 10.1136/jitc-2019-000339. PMC7174070 32217759

[123] M. Nie , L. Yang , X. Bi , et al., “Neutrophil Extracellular Traps Induced by IL8 Promote Diffuse Large B‐Cell Lymphoma Progression via the TLR9 Signaling,” Clinical Cancer Research 25, no. 6 (2019): 1867–1879, 10.1158/1078-0432.Ccr-18-1226. 30446590

[124] X. Xia , Z. Zhang , C. Zhu , et al., “Neutrophil Extracellular Traps Promote Metastasis in Gastric Cancer Patients with Postoperative Abdominal Infectious Complications,” Nature Communications 13, no. 1 (2022): 1017, 10.1038/s41467-022-28492-5. PMC8866499 35197446

[125] S.‐S. Xu , H. Li , T.‐J. Li , et al., “Neutrophil Extracellular Traps and Macrophage Extracellular Traps Predict Postoperative Recurrence in Resectable Nonfunctional Pancreatic Neuroendocrine Tumors,” Frontiers in Immunology 12 (2021): 577517, 10.3389/fimmu.2021.577517. 34084158 PMC8168461

[126] B. Muqaku , D. Pils , J. C. Mader , et al., “Neutrophil Extracellular Trap Formation Correlates with Favorable Overall Survival in High Grade Ovarian Cancer,” Cancers 12, no. 2 (2020): 505, 10.3390/cancers12020505. 32098278 PMC7072166

[127] Y. Liu and X. Cao , “Characteristics and Significance of the Pre‐Metastatic Niche,” Cancer Cell 30, no. 5 (2016): 668–681, 10.1016/j.ccell.2016.09.011. 27846389

[128] K. E. de Visser and J. A. Joyce , “The Evolving Tumor Microenvironment: From Cancer Initiation to Metastatic Outgrowth,” Cancer Cell 41, no. 3 (2023): 374–403, 10.1016/j.ccell.2023.02.016. 36917948

[129] S. Najmeh , J. Cools‐Lartigue , R. F. Rayes , et al., “Neutrophil Extracellular Traps Sequester Circulating Tumor Cells via β1‐integrin Mediated Interactions,” International Journal of Cancer 140, no. 10 (2017): 2321–2330, 10.1002/ijc.30635. 28177522

[130] Z. Liu , Z. Zhang , Y. Zhang , et al., “Spatial Transcriptomics Reveals That Metabolic Characteristics Define the Tumor Immunosuppression Microenvironment via iCAF Transformation in Oral Squamous Cell Carcinoma,” International Journal of Oral Science 16, no. 1 (2024): 9, 10.1038/s41368-023-00267-8. 38287007 PMC10824761

[131] P. C.‐T. Tang , J. Y.‐F. Chung , V. W.‐W. Xue , et al., “Smad3 Promotes Cancer‐Associated Fibroblasts Generation via Macrophage–Myofibroblast Transition,” Advanced Science 9, no. 1 (2022): 2101235, 10.1002/advs.202101235. 34791825 PMC8728853

[132] M. Zhou , B. Guan , Y. Liu , et al., “Fibrinogen‐like 2 in Tumor‐Associated Macrophage‐Derived Extracellular Vesicles Shapes an Immunosuppressive Microenvironment in Colorectal Liver Metastases by Promoting Tumor Stemness and Neutrophil Extracellular Traps Formation,” Cancer Letters 618 (2025): 217642, 10.1016/j.canlet.2025.217642. 40097065

[133] X. Zhou , C. Wu , X. Wang , et al., “Tumor Cell‐released Autophagosomes (TRAPs) Induce PD‐L1‐decorated NETs That Suppress T‐cell Function to Promote Breast Cancer Pulmonary Metastasis,” Journal for ImmunoTherapy of Cancer 12, no. 6 (2024): 009082, 10.1136/jitc-2024-009082. PMC11216055 38926151

[134] H. Wang , H. Zhang , Y. Wang , et al., “Regulatory T‐cell and Neutrophil Extracellular Trap Interaction Contributes to Carcinogenesis in Non‐alcoholic Steatohepatitis,” Journal of Hepatology 75, no. 6 (2021): 1271–1283, 10.1016/j.jhep.2021.07.032. 34363921 PMC12888775

[135] H. Zhou , C. Zhu , Q. Zhao , et al., “Wrecking Neutrophil Extracellular Traps and Antagonizing Cancer‐associated Neurotransmitters by Interpenetrating Network Hydrogels Prevent Postsurgical Cancer Relapse and Metastases,” Bioact Mater 39 (2024): 14, 10.1016/j.bioactmat.2024.05.022. 38783926 PMC11112132

[136] R. Liang , H. Lu , H. Zhu , et al., “Radiation‐primed TGF‐β Trapping by Engineered Extracellular Vesicles for Targeted Glioblastoma Therapy,” Journal of Controlled Release 370 (2024): 821–834, 10.1016/j.jconrel.2024.05.022. 38740092

[137] L. Cao , M. Shao , Y. Gu , et al., “Calceolarioside B Targets MMP12 in the Tumor Microenvironment to Inhibit M2 Macrophage Polarization and Suppress Hepatocellular Carcinoma Progression,” Phytomedicine 142 (2025): 156805, 10.1016/j.phymed.2025.156805. 40347889

[138] D. Zhu , Y. Lu , L. Gui , et al., “Self‐assembling, pH‐Responsive Nanoflowers for Inhibiting PAD4 and Neutrophil Extracellular Trap Formation and Improving the Tumor Immune Microenvironment,” Acta Pharmaceutica Sinica B 12, no. 5 (2022): 2592–2608, 10.1016/j.apsb.2021.11.006. 35646534 PMC9136569

[139] D. Zhu , Y. Lu , B. Hu , et al., “Highly‐Tumor‐Targeted PAD4 Inhibitors with PBA Modification Inhibit Tumors in Vivo by Specifically Inhibiting the PAD4‐H3cit‐NETs Pathway in Neutrophils,” European Journal of Medicinal Chemistry 258 (2023): 115619, 10.1016/j.ejmech.2023.115619. 37421890

[140] H. Zhao , L. Wu , G. Yan , et al., “Inflammation and Tumor Progression: Signaling Pathways and Targeted Intervention,” Signal Transduction and Targeted Therapy 6 (2021): 263, 10.1038/s41392-021-00658-5. 34248142 PMC8273155

[141] C. Han , V. Godfrey , Z. Liu , et al., “The AIM2 and NLRP3 Inflammasomes Trigger IL‐1–Mediated Antitumor Effects During Radiation,” Science Immunology 6 (2021), 10.1126/sciimmunol.abc6998. 33963060

[142] T. Atsumi , R. Singh , L. Sabharwal , et al., “Inflammation Amplifier, a New Paradigm in Cancer Biology,” Cancer Research 74, no. 1 (2014): 8–14, 10.1158/0008-5472.Can-13-2322. 24362915

[143] J. Zhang , J. Zhang , L. Han , et al., “Inflammation Awakens Dormant Cancer Cells by Modulating the Epithelial–Mesenchymal Phenotypic State,” Proceedings of the National Academy of Sciences U S A 122, no. 36 (2025): 2515009122, 10.1073/pnas.2515009122. PMC12435312 40901881

[144] D. E. Johnson , R. A. O'Keefe , and J. R. Grandis , “Targeting the IL‐6/JAK/STAT3 Signalling Axis in Cancer,” Nature Reviews Clinical Oncology 15, no. 4 (2018): 234–248, 10.1038/nrclinonc.2018.8. PMC5858971 29405201

[145] J. Yuan , X. Dong , J. Yap , and J. Hu , “The MAPK and AMPK Signalings: Interplay and Implication in Targeted Cancer Therapy,” Journal of Hematology & Oncology 13, no. 1 (2020): 113, 10.1186/s13045-020-00949-4. 32807225 PMC7433213

[146] M. Nair , A. Samidurai , A. Das , S. S. Kakar , and R. C. Kukreja , “Ovarian Cancer and the Heart: Pathophysiology, Chemotherapy‐induced Cardiotoxicity, and New Therapeutic Strategies,” Journal of Ovarian Research 18, no. 1 (2025): 72, 10.1186/s13048-025-01636-z. 40188339 PMC11971845

[147] A.‐L. Xia , X.‐C. Wang , Y.‐J. Lu , X.‐J. Lu , and B. Sun , “Chimeric‐Antigen Receptor T (CAR‐T) Cell Therapy for Solid Tumors: Challenges and Opportunities,” Oncotarget 8, no. 52 (2017): 90521–90531, 10.18632/oncotarget.19361. 29163850 PMC5685771

[148] R. S. Riley , C. H. June , R. Langer , and M. J. Mitchell , “Delivery Technologies for Cancer Immunotherapy,” Nature Reviews Drug Discovery 18, no. 3 (2019): 175–196, 10.1038/s41573-018-0006-z. 30622344 PMC6410566

[149] S. Zuo , J. Song , J. Zhang , Z. He , B. Sun , and J. Sun , “Nano‐Immunotherapy for Each Stage of Cancer Cellular Immunity: Which, Why, and What?,” Theranostics 11, no. 15 (2021): 7471–7487, 10.7150/thno.59953. 34158861 PMC8210608

[150] T. Tang , X. Huang , G. Zhang , Z. Hong , X. Bai , and T. Liang , “Advantages of Targeting the Tumor Immune Microenvironment over Blocking Immune Checkpoint in Cancer Immunotherapy,” Signal Transduction and Targeted Therapy 6, no. 1 (2021): 72, 10.1038/s41392-020-00449-4. 33608497 PMC7896069

[151] S. Rafiq , C. S. Hackett , and R. J. Brentjens , “Engineering Strategies to Overcome the Current Roadblocks in CAR T Cell Therapy,” Nature Reviews Clinical Oncology 17, no. 3 (2020): 147–167, 10.1038/s41571-019-0297-y. PMC7223338 31848460

[152] E. K. Moon , L.‐C. Wang , D. V. Dolfi , et al., “Multifactorial T‐cell Hypofunction That Is Reversible Can Limit the Efficacy of Chimeric Antigen Receptor–Transduced Human T cells in Solid Tumors,” Clinical Cancer Research 20, no. 16 (2014): 4262–4273, 10.1158/1078-0432.Ccr-13-2627. 24919573 PMC4134701

[153] A. Mayer and P. Vaupel , “lactate Accumulation, and Acidosis: Siblings or Accomplices Driving Tumor Progression and Resistance to Therapy?,” Advances in Experimental Medicine and Biology 789 (2013): 203, 10.1007/978-1-4614-7411-1_28. 23852496

[154] R. M. Sterner , R. Sakemura , M. J. Cox , et al., “GM‐CSF Inhibition Reduces Cytokine Release Syndrome and Neuroinflammation but Enhances CAR‐T Cell Function in Xenografts,” Blood 133, no. 7 (2019): 697–709, 10.1182/blood-2018-10-881722. 30463995 PMC6376281

[155] M. Sachdeva , P. Duchateau , S. Depil , L. Poirot , and J. Valton , “Granulocyte–Macrophage Colony‐stimulating Factor Inactivation in CAR T‐Cells Prevents Monocyte‐Dependent Release of Key Cytokine Release Syndrome Mediators,” Journal of Biological Chemistry 294, no. 14 (2019): 5430–5437, 10.1074/jbc.AC119.007558. 30804212 PMC6462525

[156] M. Kurachi , “CD8^+^ T cell exhaustion,” Seminars in Immunopathology 41, no. 3 (2019): 327–337, 10.1007/s00281-019-00744-5. 30989321

[157] S. D. Blackburn , H. Shin , W. N. Haining , et al., “Coregulation of CD8^+^ T Cell Exhaustion by Multiple Inhibitory Receptors during Chronic Viral Infection,” Nature Immunology 10, no. 1 (2009): 29–37, 10.1038/ni.1679. 19043418 PMC2605166

[158] K. Yin , M. J. Peluso , X. Luo , et al., “Long COVID Manifests with T Cell Dysregulation, Inflammation and an Uncoordinated Adaptive Immune Response to SARS‐CoV‐2,” Nature Immunology 25, no. 2 (2024): 218–225, 10.1038/s41590-023-01724-6. 38212464 PMC10834368

[159] L. J. Dooling , J. C. Andrechak , B. H. Hayes , et al., “Cooperative Phagocytosis of Solid Tumours by Macrophages Triggers Durable Anti‐Tumour Responses,” Nature Biomedical Engineering 7, no. 9 (2023): 1081–1096, 10.1038/s41551-023-01031-3. PMC10791169 37095318

[160] Y. Yang , F. Ma , Z. Liu , et al., “The ER‐Localized Ca2^+^‐Binding Protein Calreticulin Couples ER Stress to Autophagy by Associating with Microtubule‐Associated Protein 1A/1B Light Chain 3,” Journal of Biological Chemistry 294, no. 3 (2019): 772–782, 10.1074/jbc.RA118.005166. 30429217 PMC6341397

[161] M. Obeid , A. Tesniere , F. Ghiringhelli , et al., “Calreticulin Exposure Dictates the Immunogenicity of Cancer Cell Death,” Nature Medicine 13, no. 1 (2007): 54–61, 10.1038/nm1523. 17187072

[162] I. Truxova , L. Kasikova , C. Salek , et al., “Calreticulin Exposure on Malignant Blasts Correlates with Improved Natural Killer Cell‐mediated Cytotoxicity in Acute Myeloid Leukemia Patients,” Haematologica 105, no. 7 (2020): 1868–1878, 10.3324/haematol.2019.223933. 31582537 PMC7327638

[163] H. Huang , Q.‐S. Tong , J.‐Y. Zhang , et al., “Phagocytosis‐Activating Nanocomplex Orchestrates Macrophage‐Mediated Cancer Immunotherapy,” Advanced Materials 37, no. 28 (2025): 2500982, 10.1002/adma.202500982. 40289887

[164] S. Z. Shalhout , D. M. Miller , K. S. Emerick , and H. L. Kaufman , “Therapy with Oncolytic Viruses: Progress and Challenges,” Nature Reviews Clinical Oncology 20, no. 3 (2023): 160–177, 10.1038/s41571-022-00719-w. 36631681

[165] J. Kwon and S. F. Bakhoum , “The Cytosolic DNA‐Sensing cGAS–STING Pathway in Cancer,” Cancer Discovery 10, no. 1 (2020): 26–39, 10.1158/2159-8290.Cd-19-0761. 31852718 PMC7151642

[166] O. Demaria , A. De Gassart , S. Coso , et al., “STING Activation of Tumor Endothelial Cells Initiates Spontaneous and Therapeutic Antitumor Immunity,” Proceedings of the National Academy of Sciences 112, no. 50 (2015): 15408–15413, 10.1073/pnas.1512832112. PMC4687570 26607445

[167] H.‐X. Sha , Y.‐B. Liu , Y.‐L. Qiu , et al., “Neutrophil Extracellular Traps Trigger Alveolar Epithelial Cell Necroptosis through the cGAS‐STING Pathway During ACUTE Lung Injury in Mice,” International Journal of Biological Sciences 20, no. 12 (2024): 4713–4730, 10.7150/ijbs.99456. 39309425 PMC11414388

[168] P. Wang , H. Liu , L. Guo , et al., “MIL‐100(Fe)‐based Co‐delivery Platform as Cascade Synergistic Chemotherapy and Immunotherapy Agents for Colorectal Cancer via the cGAS‐STING Pathway,” Acta Biomaterialia 204 (2025): 582–595, 10.1016/j.actbio.2025.08.021. 40812610

[169] S. Li , B. Mirlekar , B. M. Johnson , et al., “STING‐induced Regulatory B Cells Compromise NK Function in Cancer Immunity,” Nature 610, no. 7931 (2022): 373–380, 10.1038/s41586-022-05254-3. 36198789 PMC9875944

[170] X. Zhang , L. Zhang , Y.‐M. Tan , et al., “Hepcidin Gene Silencing Ameliorated Inflammation and Insulin Resistance in Adipose Tissue of db/db Mice via Inhibiting METs Formation,” Molecular Immunology 133 (2021): 110–121, 10.1016/j.molimm.2021.02.015. 33640761

[171] L.‐Y. Yang , X.‐T. Shen , H.‐T. Sun , W.‐W. Zhu , J.‐B. Zhang , and L. Lu , “Neutrophil Extracellular Traps in Hepatocellular Carcinoma Are Enriched in Oxidized Mitochondrial DNA Which Is Highly Pro‐inflammatory and Pro‐metastatic,” Journal of Cancer 13, no. 4 (2022): 1261–1271, 10.7150/jca.64170. 35281873 PMC8899377

[172] M. García‐Bengoa , M. Meurer , R. Goethe , M. Singh , R. Reljic , and M. von Köckritz‐Blickwede , “Role of Phagocyte Extracellular Traps during Mycobacterium Tuberculosis Infections and Tuberculosis Disease Processes,” Frontiers in Microbiology 14 (2023): 983299, 10.3389/fmicb.2023.983299. 37492257 PMC10365110

[173] J. Ding , N. Xu , J. Wang , et al., “Plancitoxin‐1 Mediates Extracellular Trap Evasion by the Parasitic Helminth Trichinella Spiralis,” BMC Biology 22, no. 1 (2024): 158, 10.1186/s12915-024-01958-2. 39075478 PMC11287892

[174] Y. Xiao , T. Ding , H. Fang , et al., “Innovative Bio‐based Hydrogel Microspheres Micro‐Cage for Neutrophil Extracellular Traps Scavenging in Diabetic Wound Healing,” Advanced Science 11, no. 21 (2024): 2401195, 10.1002/advs.202401195. 38582501 PMC11151043

[175] Q. L. Quoc , T. B. T. Cao , J.‐Y. Moon , et al., “Contribution of Monocyte and Macrophage Extracellular Traps to Neutrophilic Airway Inflammation in Severe Asthma,” Allergology International 73, no. 1 (2024): 81–93, 10.1016/j.alit.2023.06.004. 37365039

[176] D.‐I. Cho , M. R. Kim , H.‐Y. Jeong , et al., “Mesenchymal Stem Cells Reciprocally Regulate the M1/M2 Balance in Mouse Bone Marrow‐derived Macrophages,” Experimental & Molecular Medicine 46, no. 1 (2014): 70, 10.1038/emm.2013.135. PMC3909888 24406319

[177] K. Martinod , T. Witsch , L. Erpenbeck , et al., “Peptidylarginine Deiminase 4 Promotes Age‐related Organ Fibrosis,” Journal of Experimental Medicine 214, no. 2 (2017): 439–458, 10.1084/jem.20160530. 28031479 PMC5294849

[178] G. Pironti , S. Gastaldello , D. E. Rassier , et al., “Citrullination is Linked to Reduced Ca 2^+^ Sensitivity in Hearts of a Murine Model of Rheumatoid Arthritis,” Acta Physiologica 236, no. 3 (2022): 13869, 10.1111/apha.13869. PMC9788013 36002394

[179] E. J. C. Tejeda , A. M. Bello , E. Wasilewski , A. Koebel , S. Dunn , and L. P. Kotra , “Noncovalent Protein Arginine Deiminase (PAD) Inhibitors Are Efficacious in Animal Models of Multiple Sclerosis,” Journal of Medicinal Chemistry 60, no. 21 (2017): 8876–8887, 10.1021/acs.jmedchem.7b01102. 29045782

[180] M. Alariqi , M. Ramadan , L. Yu , et al., “Enhancing Specificity, Precision, Accessibility, Flexibility, and Safety to Overcome Traditional CRISPR/Cas Editing Challenges and Shape Future Innovations,” Advanced Science 12, no. 28 (2025): 2416331, 10.1002/advs.202416331. PMC12302564 40548648

[181] Q. Shi , Q. Shen , Y. Liu , et al., “Increased Glucose Metabolism in TAMs Fuels O‐GlcNAcylation of Lysosomal Cathepsin B to Promote Cancer Metastasis and Chemoresistance,” Cancer Cell 40, no. 10 (2022): 1207–1222.e10, 10.1016/j.ccell.2022.08.012. 36084651

[182] B. Du , J. Qin , B. Lin , J. Zhang , D. Li , and M. Liu , “CAR‐T Therapy in Solid Tumors,” Cancer Cell 43, no. 4 (2025): 665–679, 10.1016/j.ccell.2025.03.019. 40233718

[183] Y. He , L. Zhan , J. Shi , et al., “The Combination of R848 with Sorafenib Enhances Antitumor Effects by Reprogramming the Tumor Immune Microenvironment and Facilitating Vascular Normalization in Hepatocellular Carcinoma,” Advanced Science 10, no. 18 (2023): 2207650, 10.1002/advs.202207650. 37083239 PMC10288281

[184] T. Kimura , Y. Kato , Y. Ozawa , et al., “Immunomodulatory Activity of Lenvatinib Contributes to Antitumor Activity in the Hepa1‐6 hepatocellular carcinoma model,” Cancer Science 109, no. 12 (2018): 3993–4002, 10.1111/cas.13806. 30447042 PMC6272102

[185] M. A. Meyer , J. M. Baer , B. L. Knolhoff , et al., “Breast and Pancreatic Cancer Interrupt IRF8‐dependent Dendritic Cell Development to Overcome Immune Surveillance,” Nature Communications 9, no. 1 (2018): 1250, 10.1038/s41467-018-03600-6. PMC5871846 29593283

[186] A. J. Giles , C. M. Reid , J. D. Evans , et al., “Activation of Hematopoietic Stem/Progenitor Cells Promotes Immunosuppression Within the Pre–metastatic Niche,” Cancer Research 76, no. 6 (2016): 1335–1347, 10.1158/0008-5472.Can-15-0204. 26719537 PMC4794356

[187] W. Liang , W. Guan , R. Chen , et al., “Cancer Patients in SARS‐CoV‐2 Infection: A Nationwide Analysis in China,” The Lancet Oncology 21, no. 3 (2020): 335–337, 10.1016/s1470-2045(20)30096-6. 32066541 PMC7159000

[188] L. H. Butterfield and Y. G. Najjar , “Immunotherapy Combination Approaches: Mechanisms, Biomarkers and Clinical Observations,” Nature Reviews Immunology 24, no. 6 (2024): 399–416, 10.1038/s41577-023-00973-8. PMC11460566 38057451

[189] Z. Chen , F. Yang , Z. Jiang , et al., “Ivonescimab plus Chemotherapy Versus Tislelizumab plus Chemotherapy as First‐line Treatment for Advanced Squamous Non‐small‐cell Lung Cancer (HARMONi‐6): A Randomised, Double‐blind, Phase 3 Trial,” The Lancet 406 (2025): 2078–2088, 10.1016/s0140-6736(25)01848-3. 41125109

[190] J. Galon and D. Bruni , “Approaches to Treat Immune Hot, Altered and Cold Tumours with Combination Immunotherapies,” Nature Reviews Drug Discovery 18, no. 3 (2019): 197–218, 10.1038/s41573-018-0007-y. 30610226

[191] B. R. Kimmel , K. Arora , N. C. Chada , et al., “Potentiating Cancer Immunotherapies with Modular Albumin‐Hitchhiking Nanobody–STING Agonist Conjugates,” Nature Biomedical Engineering 9, no. 10 (2025): 1719–1739, 10.1038/s41551-025-01400-0. PMC12532571 40500332

[192] X. Liu , Z. Cao , W. Wang , et al., “Engineered Extracellular Vesicle‐Delivered CRISPR/Cas9 for Radiotherapy Sensitization of Glioblastoma,” ACS Nano 17, no. 17 (2023): 16432–16447, 10.1021/acsnano.2c12857. 37646615 PMC10510715

[193] C. Tang , Y. Jin , M. Wu , et al., “A Biomimic Anti‐neuroinflammatory Nanoplatform for Active Neutrophil Extracellular Traps Targeting and Spinal Cord Injury therapy,” Materials Today Bio 28, (2024): 101218, 10.1016/j.mtbio.2024.101218. PMC11364920 39221206

[194] N. Shen , W. Zhao , H. Chu , et al., “Targeted Delivery and Controlled Release of Polymeric Nanomedicines for Tumor Therapy,” Fundamental Research 5, no. 4 (2025): 1349–1368, 10.1016/j.fmre.2025.01.011. 40777790 PMC12327866

[195] V. F. Gomerdinger , N. Nabar , and P. T. Hammond , “Advancing Engineering Design Strategies for Targeted Cancer Nanomedicine,” Nature Reviews Cancer 25, no. 9 (2025): 657–683, 10.1038/s41568-025-00847-2. 40751005

[196] N. Picheta , J. Piekarz , K. Danilowska , K. Szklener , and S. Mandziuk , “CAR‐T in the Treatment of Solid Tumors—A Review of Current Research and Future Perspectives,” International Journal of Molecular Sciences 26, no. 19 (2025): 9486, 10.3390/ijms26199486. 41096753 PMC12525308

[197] Y. Gao , J.‐G. Zhang , Z.‐Z. Liu , et al., “Extracellular Trap Can be Trained as a Memory Response,” Virulence 13, no. 1 (2022): 471–482, 10.1080/21505594.2022.2046950. 35254202 PMC8903778

[198] T. Gebhardt , S. L. Park , and I. A. Parish , “Stem‐Like Exhausted and Memory CD8^+^ T Cells in Cancer,” Nature Reviews Cancer 23, no. 11 (2023): 780–798, 10.1038/s41568-023-00615-0. 37821656

